# Understanding Beta-Lactam-Induced Lysis at the Single-Cell Level

**DOI:** 10.3389/fmicb.2021.712007

**Published:** 2021-07-27

**Authors:** Felix Wong, Sean Wilson, Ralf Helbig, Smitha Hegde, Olha Aftenieva, Hai Zheng, Chenli Liu, Teuta Pilizota, Ethan C. Garner, Ariel Amir, Lars D. Renner

**Affiliations:** ^1^Department of Biological Engineering, Institute for Medical Engineering & Science, Massachusetts Institute of Technology, Cambridge, MA, United States; ^2^Infectious Disease and Microbiome Program, Broad Institute of MIT and Harvard, Cambridge, MA, United States; ^3^John A. Paulson School of Engineering and Applied Sciences, Harvard University, Cambridge, MA, United States; ^4^Department of Molecular and Cellular Biology, Harvard University, Cambridge, MA, United States; ^5^Center for Systems Biology, Harvard University, Cambridge, MA, United States; ^6^Leibniz Institute of Polymer Research and the Max Bergmann Center of Biomaterials, Dresden, Germany; ^7^Centre for Synthetic and Systems Biology, Institute of Cell Biology, School of Biological Sciences, University of Edinburgh, Edinburgh, United Kingdom; ^8^CAS Key Laboratory for Quantitative Engineering Biology, Shenzhen Institute of Synthetic Biology, Shenzhen Institutes of Advanced Technology, Chinese Academy of Sciences, Shenzhen, China

**Keywords:** antibiotics, cell wall, cell mechanics, turgor pressure, MreB, mechanosensitive channels

## Abstract

Mechanical rupture, or lysis, of the cytoplasmic membrane is a common cell death pathway in bacteria occurring in response to β-lactam antibiotics. A better understanding of the cellular design principles governing the susceptibility and response of individual cells to lysis could indicate methods of potentiating β-lactam antibiotics and clarify relevant aspects of cellular physiology. Here, we take a single-cell approach to bacterial cell lysis to examine three cellular features—turgor pressure, mechanosensitive channels, and cell shape changes—that are expected to modulate lysis. We develop a mechanical model of bacterial cell lysis and experimentally analyze the dynamics of lysis in hundreds of single *Escherichia coli* cells. We find that turgor pressure is the only factor, of these three cellular features, which robustly modulates lysis. We show that mechanosensitive channels do not modulate lysis due to insufficiently fast solute outflow, and that cell shape changes result in more severe cellular lesions but do not influence the dynamics of lysis. These results inform a single-cell view of bacterial cell lysis and underscore approaches of combatting antibiotic tolerance to β-lactams aimed at targeting cellular turgor.

## 1. Introduction

Understanding how antibiotics work and how to counter antibiotic resistance are two of the most pressing questions in microbiology today. While new antibacterial therapies are still being discovered, the threat of multidrug resistance persists, and more than 35,000 people die of infections caused by antibiotic-resistant microbes each year in the U.S. alone (Centers for Disease Control and Prevention, 2019). Our modern arsenal of antibiotics has largely resulted from screens for inhibitors of bacterial growth in the 1960s (Walsh, 2003; Lewis, 2010), and comparatively few antibiotics have since been introduced (Walsh, 2003). Without the discovery of novel antibiotics, it is important to better understand how known bactericidal antibiotics kill bacteria, which could better inform methods of potentiating their lethality. In contrast to studies of antibiotic lethality that have centered on bulk culture measurements (Kohanski et al., 2010; Blair et al., 2015), here we take a single-cell approach to understanding the physical processes underlying cell death by β-lactam antibiotics, the most widely-used class of antibiotics (Bush and Bradford, 2016).

In many bacteria, the peptidoglycan (PG) cell wall confers cell shape and sustains the structural integrity of the cell. The structure of this cell wall is a partially-ordered mesh of mechanically stiff glycan strands crosslinked by peptide bonds (Höltje, 1998; Cabeen and Jacobs-Wagner, 2005; Turner et al., 2013). In Gram-negative bacteria such as *Escherichia coli*, the thin cell wall is sandwiched between the inner and outer membranes, while in Gram-positive bacteria the thicker cell wall encloses a single cytoplasmic membrane. In both Gram-negative and Gram-positive bacteria, the cell wall and membranes collectively comprise the cellular envelope. The cell envelope resists the internal turgor pressure, an outward normal force exerted on the cell envelope by the cytoplasm, and, in bacteria including *E. coli*, the cell wall is maintained by penicillin-binding proteins (PBPs) and conserved membrane proteins (Jones et al., 2001; Cabeen and Jacobs-Wagner, 2005; Paradis-Bleau et al., 2010; Typas et al., 2012; Cho et al., 2016; Meeske et al., 2016). β-lactams inhibit PBP activity and the formation of peptide crosslinks (Falconer et al., 2011; Cho et al., 2014; Qiao et al., 2017). PBP inhibition is believed to result in the formation of holes in the cell wall which destabilize the cytoplasmic membrane and drive subsequent lysis (Huang et al., 2008; Chung et al., 2009; Yao et al., 2012; Cushnie et al., 2016; Wong and Amir, 2019).

There are numerous cellular features that may influence β-lactam-induced lysis at the single-cell level. While previous studies have assumed that turgor pressure drives cellular lysis (Yao et al., 2012; Reuter et al., 2013; Wong and Amir, 2019), the turgor pressure could be regulated by cellular processes including the gating of mechanosensitive channels (MSCs) (Levina et al., 1999; Haswell et al., 2011; Reuter et al., 2013; Bialecka-Fornal et al., 2015; Chure et al., 2018), which occurs as fast as milliseconds (Boer et al., 2011; Çetiner et al., 2017). Studies of the response of cells to hypoosmotic shocks, in which the osmolarity of the environment is suddenly decreased, have indicated a typical timescale for cellular volume recovery of ~1 min (Buda et al., 2016), comparable to the timescales of β-lactam-induced lysis (Yao et al., 2012; Wong and Amir, 2019). Additionally, as β-lactams inhibit peptidoglycan cell wall synthesis, the shape of a bacterium, as determined by its cell wall, may also influence—and be influenced by—β-lactam treatment. Indeed, numerical simulations have suggested that the response of *E. coli* cell shape to vancomycin treatment in susceptible cells reveals information on cell wall architecture (Huang et al., 2008). Building on these studies, here we sought to better understand the effects of three cellular features—turgor pressure, MSCs, and cell shape changes—on β-lactam-induced lysis. In addition to informing a single-cell view of bacterial cell lysis, our findings clarify the physiological features influencing the response of bacterial cells to β-lactam antibiotics.

## 2. Results

### 2.1. Mechanics of Bacterial Cell Lysis

**Figure 1:**
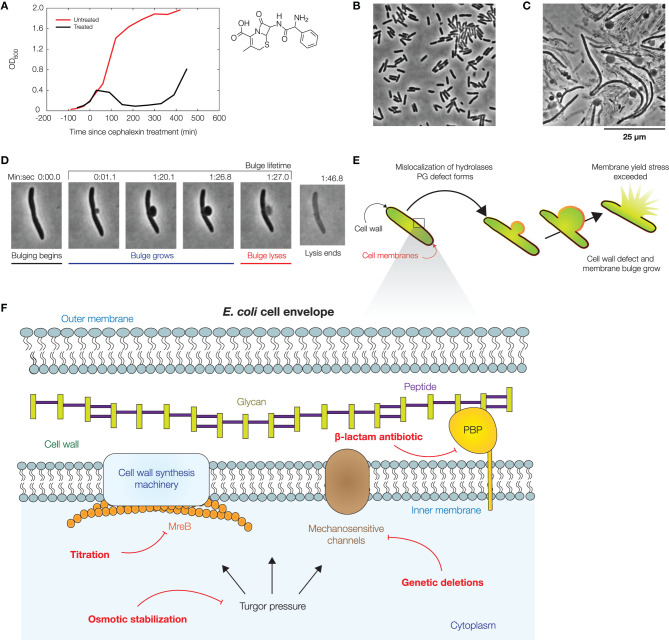
Dynamics of bacterial cell lysis. **(A)** Representative optical densities (OD_600_) of a culture of wild-type *Escherichia coli* (strain MG1655) treated by cephalexin (50 μg/mL), as shown at right. For comparison, measurements for an untreated culture are shown in red. Each curve shows one of two similar biological replicates. **(B)** Phase-contrast image of a population of *E. coli* cells immediately after cephalexin treatment. For a full timelapse, see Supplementary Movie 1. **(C)** Phase-contrast image of the same population 2 h after cephalexin treatment, illustrating that membrane bulging and lysis are common within a population. For control experiments, see Supplementary Figure 1. **(D)** Lysis dynamics of a representative log-phase *E. coli* cell during cephalexin treatment (50 μg/mL), in which the cell membrane bulges and lyses. For a full timelapse, Supplementary Movie 2. **(E)** Schematic of a mechanical model which predicts the dynamics of membrane bulging and lysis. In this model, bulging arises due to membrane reorganization and the relaxation of the cell envelope, and swelling is caused by the continued growth of cell wall defects. Lysis occurs upon reaching a yield stress in the bulge. See section 4 for details of the model. **(F)** Illustration of the *E. coli* cell envelope, with the perturbations considered in this work indicated in red.

Recent studies have characterized the dynamics of lysis in *E. coli* cells treated with β-lactams (Yao et al., 2012; Wong and Amir, 2019; Zahir et al., 2019, 2020). In previous work, Yao et al. studied the dynamics of lysis in single cells treated with cephalexin and ampicillin using high-resolution microscopy (Yao et al., 2012); here, we build on this study to explore additional perturbations involving turgor pressure, mechanosensitive channels (MSCs), and cell shape changes. Antibiotic-treated *E. coli* cells exhibit distinctive morphological features at the single-cell level, in contrast to bulk culture, in which cell death is primarily reflected by decreases in optical density (Figure 1A, Supplementary Figure 1). When treated with cephalexin, a β-lactam which inhibits cell division by blocking the activity of various PBPs including PBP3, a division-specific PBP (Curtis et al., 1979; Chung et al., 2009; Falconer et al., 2011; Kocaoglu and Carlson, 2015), cells become filamentous and typically exhibit two distinct phases. Bulging, the development of an initial membrane protrusion after ~1 h of cephalexin treatment, occurs on a timescale of seconds. Swelling, the growth of the protrusion, occurs on a timescale of minutes and is followed by explosive lysis (Figures 1B–D). These phenotypes also arise under ampicillin treatment (Supplementary Figure 1), underscoring the generality across different β-lactams. We first sought to further develop a physical model of membrane bulging and swelling (Wong and Amir, 2019) to predict the influence of turgor pressure, MCSs, and cell shape changes on lysis dynamics. The model relies on the mechanical properties of the cell envelope and coarse-grains more detailed sources of variation in the cell envelope, including lipid and peptidoglycan composition. Thus, while we detail our model for a Gram-negative bacterium here, the model can be extended to the case of a Gram-positive bacterium which has only one cell membrane, as discussed further in section 4.

We model the cell wall, inner membrane, and outer membrane of an *E. coli* cell as thin, homogeneous, elastic layers, with the inner membrane enclosing a large number of solutes which collectively and entropically generate a turgor pressure on the order of 1 atm (Koch, 1983; Cayley et al., 2000; Deng et al., 2011). Unlike the rigid cell wall, both membranes are viewed as fluid, and membrane phospholipids are assumed to rearrange around membrane-cell wall anchors. The assumption of membrane fluidity implies that the mechanical stresses in the membranes are spatially homogeneous and isotropic. The free energy of the cellular envelope and the volume it encloses comprise elastic stretching and bending terms, in addition to a pressure-volume work due to the turgor pressure (Wong and Amir, 2019). The equilibrium state of a cell, describing both the cell shape and the mechanical stresses imparted by turgor pressure inside the cellular envelope, can be found by minimizing the free energy (section 4).

Upon introducing a hole in the cell wall, minimizing the free energy predicts the formation of a partially-subtended, spherical membrane bulge; we solve the model to obtain detailed predictions in section 4 (Supplementary Figures 2–4). Importantly, the model predicts that the bulge is in equilibrium, so that the bulge stresses are *pR*/2, where *p* is the turgor pressure and *R* is the bulge radius. The model also predicts that, in the cylindrical body of the cell, the mechanical stresses in all cell envelope components sum to *pw*/4 and *pw*/2 in the axial and circumferential directions, respectively, where *w* is the rod width: here, the membranes can be load-bearing in addition to the cell wall, a prediction consistent with recent experimental and modeling studies (Hwang et al., 2018; Rojas et al., 2018; Shaevitz, 2018; Wong and Amir, 2019). For lysis to occur, the model requires that the cell membranes are sufficiently stressed. Thus, the model predicts that decreasing cellular turgor contributes to decreased mechanical stresses in the bulge and increased bulge stability. We sought to test this prediction and explore the effects of related features, including MSCs and cell shape changes, as discussed further below, experimentally (Figure 1F).

### 2.2. Osmotic Stabilization Delays, but Does Not Prevent, Lysis

**Figure 2:**
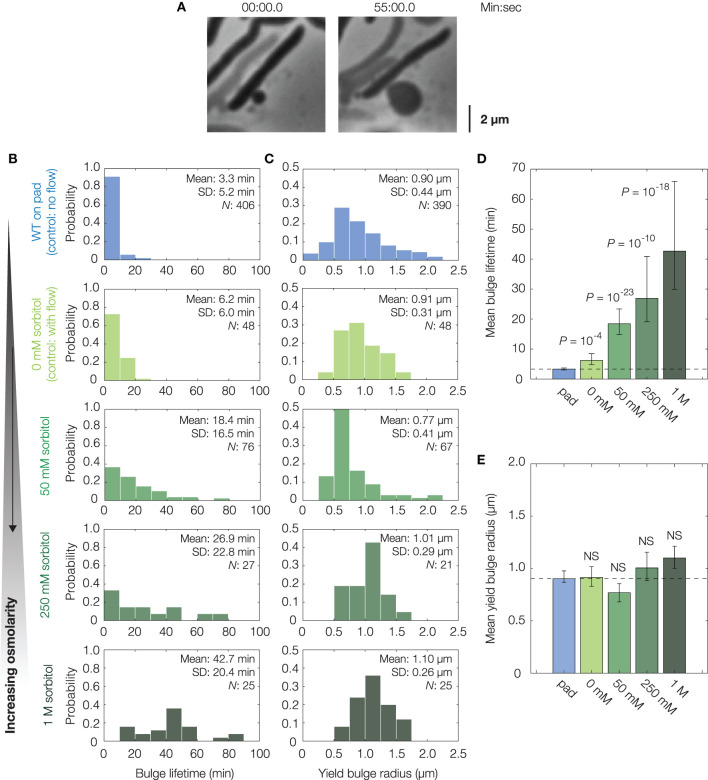
Osmotic delay of lysis in single cells. **(A)** Representative bulging *E. coli* cell (strain MG1655) osmotically stabilized by flow of hyperosmotic media (250 mM sorbitol) after cephalexin (50 μg/mL) treatment. For a full timelapse, see Supplementary Movie 3; for model predictions in cells with reduced turgor, see Supplementary Figure 2. **(B)** Distributions of bulge lifetimes for wild-type *E. coli* cells either confined to LB-agarose pads containing cephalexin (WT, on pad) or under flow of LB containing cephalexin and sorbitol (sorbitol concentrations: 0, 50, 250 mM, and 1 M). Note that WT cells in 0 mM sorbitol differ from WT cells confined to pads in that an external flow was applied in the former. Here and below, the population mean, standard deviation (SD), and number of bulged cells (*N*) are indicated. **(C)** Same as **(B)**, but for distributions of yield bulge radii. **(D)** Comparison of mean bulge lifetimes between control and osmotically shocked cells. Error bars indicate 95% confidence intervals, and *p*-values are indicated for two-sample Kolmogorov-Smirnov tests for the difference from wild-type (whose mean value is indicated by the dashed line). **(E)** Same as **(D)**, but for mean yield bulge radii. NS, not significant.

Previous studies have assumed that the lysis of bulged cells is driven by turgor pressure (Yao et al., 2012; Reuter et al., 2013; Wong and Amir, 2019), as predicted here by our model. Indeed, recent studies have shown that the osmotic stabilization of cell cultures contributes to β-lactam tolerance (Thulin et al., 2017; Mickiewicz et al., 2019). However, at the single-cell level, the turgor pressure could be regulated by MSCs and other processes on timescales comparable to, or less than, the timescales of bulging and swelling (Boer et al., 2011; Reuter et al., 2013; Bialecka-Fornal et al., 2015; Buda et al., 2016; Çetiner et al., 2017). To examine turgor pressure as a driver of lysis in single cells, we quantified two statistics—the bulge lifetime and the yield bulge radius—in our experiments lysing log-phase, wild-type cells with cephalexin treatment at a concentration of 50 μg/mL, corresponding to ~2.5× the minimum inhibitory concentration (MIC; Figures 1B–D, Supplementary Movies 1, 2, Supplementary Table 1). Here, a cell's bulge lifetime is the time between bulging and lysis given that the cell eventually lyses, and a cell's yield bulge radius indicates the final size of the bulge (Figure 1D). We next stabilized populations of bulged cells with flow of hyperosmotic media in microfluidic chambers (Figure 2A, Supplementary Movie 3).

In these experiments, growth media with the same concentration of cephalexin and varying concentrations of sorbitol, a sugar alcohol used in previous studies of osmotic shocks (Rojas et al., 2014), were introduced to populations of cells by flow at the onset of bulging (see section 4 for details). As a control, untreated cells were similarly shocked by flow of medium containing 500 mM sorbitol: such cells shrank in length by ~7%, recovered, and did not lyse, consistent with previous investigations of hyperosmotic shocks in *E. coli* (Supplementary Figure 5) (Pilizota and Shaevitz, 2012). Hyperosmotically shocking cephalexin-treated cells, we found that the lifetimes of both existing and newly-forming bulges were longer for large enough sorbitol concentrations, including those (~50 mM) corresponding to estimated values (~1 atm) of *E. coli*'s turgor pressure (Figures 2B,D) (Koch, 1983; Cayley et al., 2000; Deng et al., 2011). In particular, cells under hyperosmotic shock typically persisted for tens of minutes after bulging—a timescale comparable to the half-life of cephalexin in humans (Gower and Dash, 1969)—in contrast to ~3 min for non-osmotically shocked cells (Figure 2D). Furthermore, we observed a statistically significant increase in bulge lifetime even with flow of 0 mM sorbitol, an effect which arises because bulges can be detached from cells by the flow and remain stable without further growth, as observed empirically (Supplementary Figure 5). Although the fractions of bulged cells remained similar across all conditions involving osmotic shocks (Supplementary Figure 6), the addition of hyperosmotic media and ensuing longer bulge lifetimes of osmotically-shocked cells correlate with increased survival rates of single cells (Supplementary Figure 1). Thus, these findings are consistent with the hypothesis that turgor pressure is crucial to lysis. Consequently, they support modulation of cellular turgor as a process that can result in β-lactam tolerance. This process does not depend on changes to the MIC (Supplementary Table 1); rather, it depends on the phenotypic response of cells.

While flow of hyperosmotic media delayed lysis, we also observed that cells ultimately lysed. For long enough times, this lysis occured irrespective of the external osmolarity and therefore is not likely to arise from biased sampling (Supplementary Figure 6). Furthermore, measurements showing similar bulge radii at lysis (Figures 2C,E) suggest that lysis may occur due to recovery of cellular turgor, as the model predicts that the bulge stresses σ = *p*(*t*)*R*(*t*)/2 must increase for lysis to occur. Thus, consistent with the minute-timescale recovery of cellular turgor in response to hyperosmotic shock (Pilizota and Shaevitz, 2014), regulatory processes appear to restore the turgor pressure and eventually cause lysis.

### 2.3. Mechanosensitive Channels Fail to Protect Against Lysis

**Figure 3:**
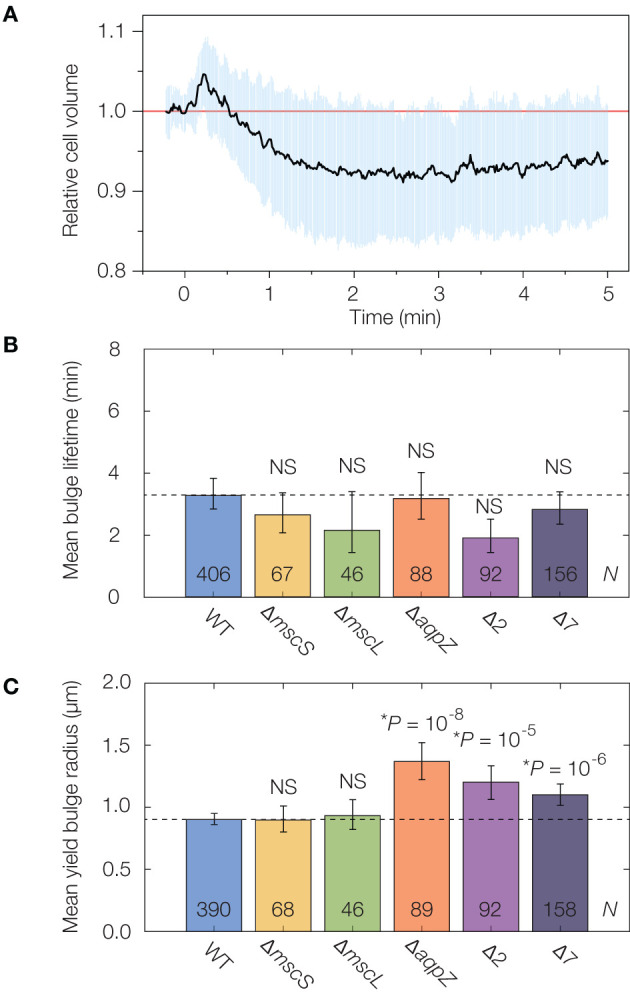
Mechanosensitive channels fail to delay or stabilize cells against lysis. **(A)** Averaged single-cell volume response of 30 wild-type (parent strain BW25113) cells to a hypoosmotic shock of 600 mM sorbitol at time *t* = 0 min, indicating volume recovery in ~30 s. Similar traces for the Δ7 strain for an osmotic shock of a similar magnitude show no volume recovery, as detailed in Supplementary Figure 7. The blue shaded region indicates one standard deviation. **(B)** Comparison of mean bulge lifetimes between WT cells and MSC mutants. Error bars indicate 95% confidence intervals, and *p*-values are indicated for a two-sample Kolmogorov-Smirnov test for the difference from wild-type (dashed line). The number of bulged cells (*N*) is indicated on each bar. For detailed histograms, see Supplementary Figure 7; for full timelapses of lysing populations, see Supplementary Movies 4, 5. NS, not significant. **(C)** Same as **(B)**, but for mean yield bulge radii. ^*^While the yield bulge radii for the Δ*aqpZ*, Δ2, and Δ7 strains differ from WT, we note they are not largely increased.

As turgor pressure appears to be re-established for lysis in osmotically shocked cells, we asked whether physiological mechanisms such as osmoregulation through MSCs could affect lysis dynamics. It has been shown that MSCs are crucial for preventing lysis in various environments, such as those involving osmotic downshifts and variations in membrane tension (Levina et al., 1999; Haswell et al., 2011; Reuter et al., 2013; Bialecka-Fornal et al., 2015; Chure et al., 2018), and prior studies have suggested that MSCs gate as fast as milliseconds (Boer et al., 2011; Çetiner et al., 2017). Experiments examining the response of MSCs to hypoosmotic shocks reveal a typical timescale for cellular volume recovery of ~1 min (Buda et al., 2016). Consistent with this study, after application of a 600 mM hypoosmotic shock, we observed both volume recovery on a timescale of ~30 s and a characteristic overshooting (Buda et al., 2016) on a timescale comparable to 1 min in wild-type cells (Figure 3A, Supplementary Figure 7). Additionally, quantitative estimates of membrane tension suggest that its increase (~11 mN/m) is sufficient to trigger MSCs in many bulged cells, as typical gating tensions are 5–15 mN/m (Buda et al., 2016)—a range less than, or comparable to, typical estimated lytic tensions of 10–20 mN/m, corresponding to membrane yield strains of ~10% (Li et al., 2013; Chabanon et al., 2017; Wong and Amir, 2019) and membrane area stretch moduli of 0.1–0.2 N/m (Sun et al., 2014) (see section 4 for additional details). Such a response could therefore contribute to bulge stability and underlie a role of MSCs in resisting the lysis we observed. However, the role of MSCs in β-lactam-mediated lysis does not seem to have been studied previously.

To probe the effects of individual MSCs on lysis, we lysed genetic knockouts of the MSC of small conductance (MscS), the MSC of large conductance (MscL), and an aquaporin (AqpZ) from the Keio collection of single knockout strains (Baba et al., 2006). As we found that the cephalexin MICs for all strains are similar to that of wild-type cells (Supplementary Table 1), we used an identical concentration of 50 μg/mL as above. We observed that the lysis dynamics of all strains were largely similar to that of wild-type. We found statistically significant differences in the yield bulge radii of the Δ*aqpZ* strain, and cannot rule out the possibility that AqpZ may influence the dynamics of β-lactam-induced lysis (Supplementary Movie 4). Nevertheless, the average bulge lifetimes and sizes are within a two-fold range in all single knockouts (Figures 3B,C, Supplementary Figure 7), suggesting that these individual MSCs do not substantially protect against lysis.

We next asked whether, instead of any single MSC, the collective action of several MSCs elicited a stronger response, as is the case when cells are hypoosmotically shocked (Buda et al., 2016). To address this question, we interrogated the recently constructed “Δ2” and “Δ7” strains of *E. coli* in which two (Δ2) and all (Δ7) the major MSCs are genetically deleted (Buda et al., 2016; Hegde, 2020). The deleted channels comprise MscS and MscL (Δ2), the MSC of miniconductance (MscM), the potassium-dependent MSC (MscK), and three MscS homologs (YnaI, YbiO, and YbdG) (Edwards et al., 2012). We validated that the channels function in wild-type cells by examining traces of volume recovery in response to hypoosmotic shocks (Figure 3A, Supplementary Figure 7). Intriguingly, we found that the lysis dynamics of both the Δ2 and Δ7 strains were quantitatively similar to that of wild-type (Figures 3B,C, Supplementary Figure 7, Supplementary Movie 5). As with the single knockouts, Δ2 and Δ7 cells exhibited bulge lifetimes and sizes approximately equal to those of wild-type cells (Figures 3B,C), and, as explained above, our model suggests that the similar bulge sizes at lysis imply that these cells have similar turgor pressures. Thus, while studies have shown the importance of MSCs in relieving membrane tension and responding to osmotic shifts (Levina et al., 1999; Haswell et al., 2011; Reuter et al., 2013; Bialecka-Fornal et al., 2015; Chure et al., 2018), our observations suggest that MSCs fail to protect against membrane bulging and lysis.

To better understand the apparent failure of MSCs to protect against lysis, we extended our model of bacterial cell lysis to account for the gating of MSCs and the transport of solutes. As detailed in section 4, we assume MSC gating to be well described by the addition of nanoscale gaps in the inner membrane (Naismith and Booth, 2012; Buda et al., 2016). We modeled the laminar outflow of intracellular solutes to the external milieu and calculated the mechanical stresses in a bulged cell as a function of time after MSC gating (Supplementary Figures 3, 4). Consistent with our experimental observations, the model predicts that the decrease in turgor elicited by MSC gating is insufficient to overcome the increase in membrane stresses due to bulge growth (Supplementary Figure 4). Namely, while solute outflow through MSCs substantially decreases the membrane tension in an unbulged cell, in a bulged cell the membrane bulge is unsupported by the cell wall and growing in time. The bulge stresses of σ = *p*(*t*)*R*(*t*)/2 are therefore carried by the membranes and increase in time due to the dependence on *R*(*t*). We find that this increase counteracts the decreases in membrane tension arising from solute outflow through MSCs (Supplementary Figure 4). Thus, the combination of our experimental observations and our biophysical model of solute transport indicate that MSCs, even when gated, can be insufficient to resist lysis.

### 2.4. Altering *E. coli* Cell Width Changes Cell Shape, but Not Susceptibility to Lysis

Finally, we sought to use our model to predict whether cell shape changes may affect lysis. As detailed in section 4, our model predicts wider cells to yield larger bulges due to the energetic trade-offs between mechanical stresses in the cylindrical bulk of the cell and stresses in the bulge (see section 4 for details). We therefore sought to experimentally test this prediction by generating *E. coli* cells of different widths.

**Figure 4:**
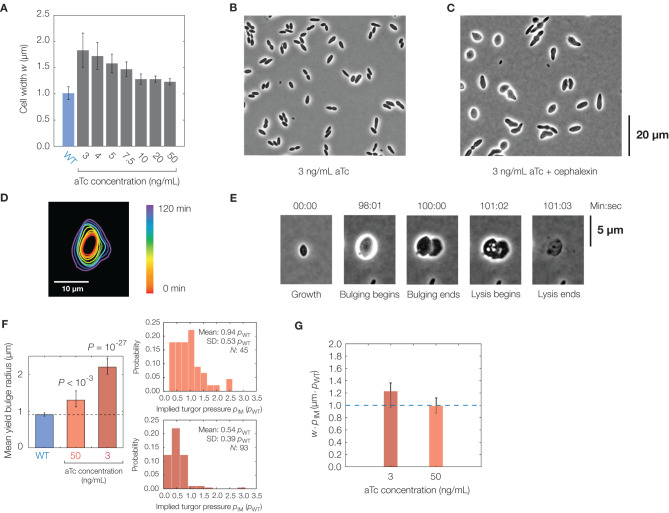
Effects of cell shape changes on lysis. **(A)** Plot of mean cell width against aTc concentration in the *mreB*-titratable strain (strain ZH1) and for wild-type (parent strain AMB1655) cells. Error bars indicate one standard deviation, and each bar represents at least 100 cells. **(B)** Phase-contrast image of a population of *mreB*-titratable cells, at an aTc concentration of 3 ng/mL, without cephalexin treatment. **(C)** Same as **(B)**, but for 2 h after cephalexin treatment, showing that most cells lose their rod shapes. **(D)** Contour image of a representative *mreB*-titratable cell, at an aTc concentration of 3 ng/mL, which does not bulge or lyse over 2 h. **(E)** Lysis dynamics of a representative *mreB*-titratable cell, at an aTc concentration of 3 ng/mL, during cephalexin treatment, with labeling corresponding to Figure 1D. For a full timelapse of a lysing population, see Supplementary Movie 6. **(F)** Comparison of mean yield bulge radii between WT and *mreB*-titratable cells. Error bars indicate 95% confidence intervals, and *p*-values refer to two-sample Kolmogolrov-Smirnov tests for the difference from wild-type (dashed line). The accompanying distributions of implied turgor pressures *p*_IM_, expressed in units of turgor pressure in wild-type cells, *p*_WT_, is shown at right. See section 2 for details of the calculation. **(G)** Plot of the product of cell width, *w*, and implied turgor pressure, *p*_IM_, against aTc concentration in the *mreB*-titratable strain. Error bars represent 95% confidence intervals, and each point represents calculations based on measurements from at least 45 cells. The blue dashed line, shown for comparison, represents the value of *w* · *p*_IM_ expected for a wild-type cell with a cell width of 1 μm.

To modulate cell width, we used an *mreB*-titratable strain of *E. coli* we previously constructed (Zheng et al., 2016). MreB is an actin homolog crucial to the cell wall synthesis required for rod shape (Jones et al., 2001; Domínguez-Escobar et al., 2011; Garner et al., 2011; van Teeffelen et al., 2011; Hussain et al., 2018; Wong et al., 2019) whose inhibition by a small molecule, A22, results in cell wall weakening and eventual lysis under typical growth conditions (Gitai et al., 2005; Bean et al., 2009; Wang et al., 2010; Furchtgott et al., 2011; Tuson et al., 2012). In the *mreB*-titratable strain, the expression of *mreB* is controlled by an inducer, anhydrotetracycline (aTc), of a *P*_tet_-*tetR* feedback loop, while the native copy of *mreB* was removed. Above a threshold concentration of 1 ng/mL aTc, decreasing aTc concentration increases cell width (Figure 4A) while leaving the growth rate unchanged, as previously described (Zheng et al., 2016).

In the *mreB*-titratable strain, we found that the cephalexin MIC was similar to that of wild-type cells (Supplementary Table 1), and hence, we induced lysis as above with cephalexin at the same concentration of 50 μg/mL. We observed that cells treated with cephalexin lost shape before membrane bulging (Figures 4B,C). These “lemon-shaped” cells exhibited varying widths along the cellular long axis over a range of aTc concentrations, with larger widths and greater heterogeneity at smaller concentrations of aTc. Regions of large widths, apparently positioned at septa, were commonly flanked by narrower cross-sections and resemble *Bacillus subtilis* cells with perturbed PG precursor pathways (Peters et al., 2016; Zhao et al., 2016). Intriguingly, and in contrast to wild-type cells, growth became isotropic in cells with severe width heterogeneity (Figure 4D), an observation which could result from misinsertion of glycan strands during PG synthesis (Hussain et al., 2018) and severely disordered wall architecture (Dion et al., 2019).

Quantifying the lysis dynamics of *mreB*-titratable cells, we found that these cells lysed similarly to wild-type cells, with membrane bulging and lysis occurring on characteristic timescales of seconds and minutes, respectively, approximately 1 h after antibiotic treatment (Figure 4E, Supplementary Figure 8, Supplementary Movie 6). Consistent with model predictions, decreasing aTc concentration correlated with larger cell widths and increased bulge radii (Figure 4F). Yet, we found that cells with larger bulges did not lyse sooner (Supplementary Figure 8), as would be expected from our model if the turgor pressure were similar across cells of different cell widths. Measurements of the bulge radii at lysis of *mreB*-titratable cells in 3 ng/mL aTc further revealed that yield bulge radii were, on average, twice as large as that of wild-type cells (Figure 4F). As we expect that the membrane composition, and hence membrane yield strain, are similar across cells, this observation suggests that the turgor pressure of *mreB*-titratable cells in the presence of 3 ng/mL aTc is, on average, half the turgor pressure of wild-type cells at lysis (Figure 4F). Intriguingly, this observation suggests that, in unbulged *mreB*-titratable cells, the mechanical stresses (proportional to *pw*) in the cell envelope remain approximately constant across different cell widths, *w* (Figure 4G).

In sum, these results reveal the effect of cell shape changes on lysis and offer biological insight into cell width maintenance. Our observations suggest that cell width changes in growing *mreB*-titratable cells may not be explained by differences in turgor pressure alone: *mreB*-titratable cells do not increase their widths by increasing turgor pressure and thus cell wall strain, as our measurements suggest that the turgor pressure is *decreased* in wider cells. Rather, both turgor pressure and cell wall synthesis may be modulated to generate cells of different widths, and our results provide evidence that the mechanical stresses in the cell envelope are regulated during the normal growth of cells. Indeed, the response of cells to osmotic stress, both hypoosmotic and hyperosmotic, has been appreciated as physiologically relevant in other contexts (Sleator and Hill, 2002), including the conversion of walled cells to wall-less L-forms (Ramijan et al., 2018; Claessen and Errington, 2019; Mickiewicz et al., 2019; Osawa and Erickson, 2019; Chikada et al., 2021). We anticipate future experiments, for instance those involving osmotic shocks, to further validate the hypothesis that the mechanical stresses in cells of different widths are approximately constant.

## 3. Discussion

Here, we have taken a single-cell approach to quantifying bacterial cell lysis across hundreds of *E. coli* cells under different physical, genetic, and physiological perturbations. We further developed a biophysical model which explains how lysis emerges as a mechanical response and suggests that the gating of MSCs are insufficient to resist lysis. Experimental results suggest that *E. coli* cells re-establish their turgor pressure irrespective of lysis and that MSCs, though active, are insufficient to prevent lysis. Furthermore, our experiments involving cell shape changes suggest that cells may regulate mechanical stresses in their cell envelopes during normal growth, and that variation in cell width does not affect the dynamics of lysis. Taken together, these results suggest that MSCs and MreB do not significantly affect the dynamics of lysis underlying β-lactam killing. In contrast, our work indicates that combination approaches which increase cellular turgor, such as jointly treating cells with hypoosmotic shocks, may be particularly effective in countering phenotypic tolerance to β-lactams. As bacterial growth quickly resumes in antibiotic-treated, osmotically-stabilized cultures (Supplementary Figure 1), our study further indicates that changes in the osmolarity of cellular environments can have clinical implications on the efficacy of β-lactams. While this notion has been appreciated in previous studies (Thulin et al., 2017; Mickiewicz et al., 2019), the present study underscores its single-cell basis.

More generally, our study demonstrates how combining theoretical modeling with physical, genetic, and physiological perturbations can reveal insight into the processes governing bacterial cell lysis. This approach to interrogating living cells may be broadly applicable for systems-level analyses of bacterial growth and bacterial stress responses, for which many molecular details remain obscure. We expect future studies to explore other cell death pathways, such as those induced by other antibiotics, at the single-cell level.

## 4. Methods

### 4.1. Bacterial Strains

For the convenience of readers, we have listed the genotypes and sources of all strains used in this study in Supplementary Table 1. The wild-type strain of *E. coli* largely used in this study is MG1655, and we verified that the morphological dynamics are statistically indistinguishable in three other wild-type-like strains, JOE309, BW25113, W3110. Strains from the Keio collection of *E. coli* single knockouts, JW2891-2 (Δ*mscS*), JW3252-1 (Δ*mscL*), and JW0859-5 (Δ*aqpZ*) have previously been described (Baba et al., 2006). These strains were verified by PCR and grown in the presence of 25 μg/mL kanamycin for selection. The Δ2 and Δ7 strains of *E. coli* have been described in previous work by the Pilizota lab (Buda et al., 2016; Hegde, 2020). These strains were constructed by knocking out up to a total of seven MSCs found in *E. coli* (Schumann et al., 2010) and grown in the presence of no antibiotics. We verified their genotypes using PCR. The *mreB*-titratable strain of *E. coli* has been described in previous work by the Liu lab (Zheng et al., 2016). In this strain, an *amp-P*_tet_*-tetR-mreB* element was inserted into the chromosomal *attB* site of *E. coli* K12 AMB1655 and the native copy of *mreB* was replaced by a kanamycin-resistance gene. The strain was cultured in the presence of 50 μg/mL ampicillin, 25 μg/mL kanamycin, and 50 ng/mL aTc for plasmid selection.

### 4.2. Bacterial Culture and Growth

Cells were grown at 37°C in liquid LB (Bertani, 1951, 2004) (LB: 10 g/L tryptone, 5 g/L yeast extract, 10 g/L NaCl) and, if required, supplemented with appropriate antibiotics. LB media containing 1.5% Difco agar (w/v), supplemented with appropriate antibiotics, was used to grow individual colonies. Tryptone, yeast extract, peptone, Petri dishes, and bacteriological agar were from Becton Dickinson (Sparks, MD) and sodium chloride was from Fisher Scientific (Fairlawn, NJ). Cells were grown from a single colony in LB, supplemented with appropriate antibiotics if required, at 37°C in 14 mL Falcon tubes (Corning, NY) and placed in a roller drum agitating at 60 rpm overnight. The overnight culture was then diluted 1:100 in fresh LB (with aTc at an appropriate concentration for *mreB*-titratable cells), and cells were allowed to grow in the same conditions for ~2 h to an optical density (OD_600_) in the range of 0.1–0.3, as measured in 2 mL working volumes using a Biowave Cell Density Meter CO8000 (VWR, Radnor, PA). Cells were then concentrated once by centrifugation at 3,000 rpm for 5 min, the supernatant was discarded, and cells were resuspended. For experiments involving agarose pads, we placed 1–2 μL of the concentrated bacterial culture on No. 1.5 coverslips (VWR, Radnor, PA) and immediately placed a 1 mm thick LB agarose (1.5%) pad on top. Cells were imaged directly afterward, so that the total time from taking cells out of culture and start of imaging was ~5 min. As we have done before for spheroplast formation (Renner and Weibel, 2011; Renner, 2019) and lysis (Wong and Amir, 2019), we treated cells with the β-lactam antibiotic cephalexin. Cephalexin hydrate (Sigma-Aldrich, St. Louis, MO) was dissolved in 1 M ammonium hydroxide stock solution. Freshly-prepared cephalexin (final concentration 50 μg/mL) and, when appropriate, aTc (Sigma-Aldrich, St. Louis, MO), were mixed with LB agarose melt before gelation—that is, at a temperature of approximately 55°C—to the final concentrations indicated. Cephalexin (final concentration 50 μg/mL) and, when appropriate, sorbitol, were added to liquid LB for microfluidic experiments, as detailed below. Note that, for the concentrations of sorbitol considered in this work, the osmolarity of the solution scales linearly with the osmolality (Rojas et al., 2014). Furthermore, while sorbitol can be metabolized by *E. coli* (Aidelberg et al., 2014), we note here that addition of sorbitol at the concentrations considered serves mainly to increase the total external media concentration, consistent with previous work by others (Rojas et al., 2014).

### 4.3. Microfluidics

For osmotic stabilization experiments, we used both custom-made and commercially-available setups. Briefly, we used the commercially-available CellASIC ONIX2 Microfluidic System (Merck, Germany) as follows. Bacteria were grown by diluting an overnight culture 1:200 in fresh LB to an OD_600_ of between 0.1 and 0.2 and incubating at 37°C. The bacterial solution was loaded into the appropriate channels using the manufacturer's pre-set loading sequence. After loading, the solution was immediately exchanged to LB+cephalexin (50 μg/mL) to induce cell lysis. At the onset of bulging, the LB+cephalexin solution was switched to LB+cephalexin+sorbitol to stabilize bulges, and the channels were continuously supplied with fresh LB+cephalexin+sorbitol at a flow rate of ~0.2 μL/h (corresponding to a set pressure of 0.5 kPa in the CellASIC system).

We also used simple, custom-made microfluidic setups comprising rectangular channels with lowered centers. These devices were designed in AutoCAD (Autodesk, San Rafael, CA), fabricated using in-house UV lithography, and replicated in polydimethylsiloxane (PDMS) by soft lithography (Weibel et al., 2007), as described previously (Renner and Weibel, 2011).

### 4.4. Microscopy

We used a Nikon Eclipse Ti inverted microscope (Nikon, Tokyo, Japan) with an enclosing custom-made incubation chamber and equipped with a 6.5 μm-pixel Hamamatsu ORCA-Flash4.0 V2 sCMOS camera (Hamamatsu, Hamamatsu City, Japan) and a Nikon Plan Apo λ 100x/1.45NA objective (Nikon, Tokyo, Japan) for imaging. We also used a Zeiss Axiovert 200 inverted microscope (Zeiss, Jena, Germany) with an enclosing custom-made incubation chamber and equipped with an Axiocam 503 mono CCD (Zeiss, Jena, Germany) and a Zeiss EC Plan-Neofluar 40x/0.75NA objective (Zeiss, Jena, Germany). All cells were imaged at 37°C inside the custom-made incubation chambers. The time between each frame during timelapse measurements ranged from 5 ms to 2 s, and the duration of timelapses varied from 10 min to 3 h. Images were recorded using NIS-Elements (Nikon, Tokyo, Japan) and AxioVision (v.4.8, Zeiss, Jena, Germany). We used ImageJ (NIH, Bethesda, MD) for cropping timelapses and the StackReg plugin (Thévenaz et al., 1998), which recursively aligns images in a sequence with geometric transformations, to correct for microscope drift as necessary. All microscopy experiments were performed independently on these two different imaging setups and replicated at least twice on each setup.

### 4.5. Bulk Culture Measurements

We verified similar growth of all strains by measuring growth curves, as shown in Supplementary Figure 1. Briefly, cells were diluted 1:100 in fresh LB from an overnight culture into 14 mL Falcon tubes and allowed to grow to an OD_600_ of ~0.2 in the growth conditions described above, with the appropriate phenotype induced for *mreB*-titratable cells. Cephalexin (final concentration 50 μg/mL) was then added, in addition to appropriate concentrations of sorbitol for osmotic shock experiments only. The OD_600_ was measured at various time points. The experiment was independently performed in 96-well plates using a Tecan Genios plate reader (Tecan, Switzerland) and a total volume of 250 μL per well to confirm the qualitative behaviors of the curves shown in Supplementary Figure 1. Each experiment was performed at least in biological duplicate.

### 4.6. Image Analysis

Lysis dynamics were annotated manually in ImageJ (National Institutes of Health, Bethesda, MD). We analyzed a total of 463 wild-type (MG1655, JOE309, BW25113, W3110) cells in control (agarose pad) experiments, 49 wild-type (MG1655) cells in control flow experiments, 77 wild-type (MG1655) cells in flow experiments at a sorbitol concentration of 50 mM, 27 wild-type (MG1655) cells in flow experiments at a sorbitol concentration of 250 mM, 80 Δ*mscS* cells, 60 Δ*mscL* cells, 109 Δ*aqpZ* cells, 125 Δ2 cells, 201 Δ7 cells, 85 *mreB*-titratable cells at an aTc concentration of 50 ng/mL, and 212 *mreB*-titratable cells at an aTc concentration of 3 ng/mL. Experiments were not randomized, and we were not blinded to allocation during experiments and assessment of results. All cells for which we could resolve bulging and lysis were used.

The shapes of individual cells were annotated with subpixel resolution as follows. Bulged cells were fit to cylinders with protruding spheres to determine bulge radii and defect lengths (taken here to be the neck-to-neck lengths of bulges). The bulge lifetime was determined as the time in which a bulge of radius larger than a predetermined threshold value, 0.2 μm, appeared and lysed. In the rare case that cells exhibited multiple bulges, we annotated only the bulge which lysed first. To account only for bulging cells, we removed from the data all cells which bulged and lysed within a single timestep of our imaging or 1 s, whichever was larger. We also removed from the data all cells in which the bulge dimensions could not be visually determined, often because the bulge was occluded or moved out of the imaging plane. For each bulge, the bulge radius at lysis was fit in the frame immediately before lysis. The yield defect length was also measured for this frame. For Figure 4F of the main text, the implied turgor pressure was calculated as *p*_IM_ = *p*_WT_〈*R*_WT_〉/*R*_*mreB*_, where *p*_WT_ is the turgor pressure of a wild-type (WT) cell, assumed to be constant over cells, 〈*R*_WT_〉 is the yield bulge radius of wild-type cells in control experiments, averaged over the entire population, and *R*_*mreB*_ is the yield bulge radius of *mreB*-titratable cells. The standard deviations in Figure 4G of the main text were calculated assuming all variables to be statistically independent and using the variance relation Var(*XY*) = Var(*X*)Var(*Y*) + Var(*X*)〈*Y*〉^2^ + Var(*Y*)〈*X*〉^2^, where *X* and *Y* are random variables and 〈·〉 denotes their means (Goodman, 1960).

### 4.7. Cell Volume Dynamics

In Supplementary Figure 7A, *E. coli* Δ7 cells with pWR21 were grown in modified M9 (MM9) media, in which the potassium phosphate salts in the regular M9 salts were replaced with sodium phosphate salts (Harbor, 2010), and supplemented with 0.3% glucose, minimal essential amino acids, and an additional 300 mM NaCl. pWR21 contained constitutively expressing cytoplasmic eGFP that was used for cell cytoplasmic volume measurement (Pilizota and Shaevitz, 2012). Cells were cultured at 37°C to an OD_600_ of 0.3–0.5 aerobically with shaking at 220 rpm. Cells were next attached to a microscope tunnel slide using poly-L-lysine as previously described (Buda et al., 2016). Cells were then imaged with an epifluorescence microscope (Nikon CFI Plan Apochromat λ 100x/1.45NA objective, Nikon, Tokyo, Japan) at 21°C, and the field of view was stabilized using back-focal-plane inferometry (Pilizota and Shaevitz, 2012). A 300 mM (460 mOsmol) hypoosmotic shock was delivered by flushing with 35 μL MM9 media with no additional NaCl, and the tunnel ends were sealed with a liquid sealant (CoverGrip TM Coverslip Sealant, Biotium, Fremont, CA) to avoid drying of the slide. Images were captured every 0.33 s with excitation at 500 nm and emission at 515 nm. Cells uniformly attached to the coverslip were selected for analysis. For Figure 3A, the same protocol was repeated with *E. coli* BW25113 in LB media—the same media used in all lysis experiments—and 600 mM sorbitol. We note that previously, we observed at the single-cell level that upon downshock, cells expand quickly (several seconds) and continue to recover on a timescale of tens of seconds (Buda et al., 2016). In this previous study, cells were grown in M9 medium as described in the preceding paragraph and supplemented with NaCl to increase the osmolarity. Despite the difference in growth condition and the solute used to increase the osmolarity between Buda et al. (2016) and Figure 3A, the response we observed to the downshock is similar. This is expected as the downshock is a passive response. Supplementary Figure 7 shows the Δ7 strain subjected to a similar downshock magnitude as in Figure 3A and in the same growth condition as in previous work (Buda et al., 2016); for comparison, wild-type traces from previous work (Buda et al., 2016) are included in Supplementary Figure 7.

The cell volume dynamics during osmotic downshocks were analyzed as previously described (Pilizota and Shaevitz, 2012; Buda et al., 2016). Briefly, cytoplasmic fluorescent protein was used to mark the cell and the total number of pixels whose intensity was above a selected normalized threshold value was counted. Individual cell volume traces were normalized by the average volume calculated from the first ten time points.

### 4.8. Statistical Testing

The confidence intervals for all bulge parameters in this work were calculated by bootstrapping with 10,000 subsamples using MATLAB's bootci function (Supplementary Table 3). We note here that we also found that the distributions of bulge radii and bulge lifetimes could usually be described by the lognormal and exponential distributions, as verified using a χ^2^ goodness-of-fit test at a standard α = 0.01 significance level; for most conditions, we found that the test result does not reject the null hypothesis that the data come from the corresponding distributions (Supplementary Table 3). Unless otherwise specified, in order to compare samples from different conditions without assuming specific underlying distributions, two-sample Kolmogorov-Smirnov tests were performed for all datasets shown in the main text at the α = 0.001 significance level as to reduce the likelihood of Type I (false positive) error. As *mreB*-titratable cells differ in shape with and without cephalexin, we bootstrapped the cell width statistics (*w*) of Figure 4A to calculate confidence intervals for Figure 4G of the main text.

### 4.9. MIC Determination

We determined cephalexin MICs for all strains considered in this work by inoculating a 1:10,000 dilution of an overnight culture into fresh LB in 96-well plates, in working volumes of 200 μL, with 2-fold dilutions of antibiotic across wells. The MIC was determined as the minimum concentration at which no visible growth occurred overnight (OD_600_ < 0.1). A summary of all MIC values thus determined is provided in Supplementary Table 1, and all measurements were performed in biological duplicate.

### 4.10. Mechanical Model of Bacterial Cell Lysis

#### 4.10.1. The Model

This work builds on a model of bacterial cell lysis introduced by some of us (Wong and Amir, 2019), showing how it can be extended to yield predictions for the perturbations considered in this work. Our model is different from a previous model by Daly et al. (2011) due to our focus on stretching energies, and not bending energies, as the main source of strain in the cell envelope; additional comparisons between these models are detailed in Wong and Amir (2019). Here, for completeness, the model is described in full, then extended. We model the cell wall, inner membrane, and outer membrane of a Gram-negative cell as thin, homogeneous, elastic layers in contact. Unlike the rigid cell wall, the membranes are fluid and hence free to change their reference configurations under the constraint of fixed reference areas. The free energy of the cellular envelope and the volume it encloses is

(1) $$\begin{array}{l} F=E_{\text{strain}}^{w}+E_{\text{strain}}^{i}+E_{\text{strain}}^{o}-TS, \end{array}$$

where the superscripts ^*w*^, ^*i*^, and ^*o*^ denote cell wall, inner membrane, and outer membrane quantities, respectively, *E*_strain_ is the elastic strain energy, *T* is the temperature, and *S* is the entropy of mixing water and solutes. Assuming only water molecules outside the cell for simplicity, *S* = −*k*(*n*_*s*_ ln *x*_*s*_ + *n*_*w*_ ln *x*_*w*_), where *k* is Boltzmann's constant, *x*_*s*_ and *x*_*w*_ are the number fractions of solute and water molecules, respectively, and *n*_*s*_ and *n*_*w*_ are the numbers of solute and water molecules, respectively. Note that the turgor pressure is defined as *p* = *kTC*, where *C* is the solute concentration, and that the origin of turgor is entropic. Furthermore, we do not consider growth of the cell due to the timescales of interest in this work. Below, we neglect the hemispherical poles of the cell for simplicity and consider only the cylindrical bulk.

To describe how the cell envelope reacts to the turgor pressure, we first note that, for characteristic parameter values relevant to *E. coli* (Supplementary Table 2), the stretching energy will dominate the bending energy in *E*_strain_, as is typical for thin shells. We assume an orthotropic constitutive relation for the cell wall, consistent with evidence for a larger Young's modulus in the circumferential direction than that in the axial direction (Lan et al., 2007; Deng et al., 2011), but note that the main predictions relevant to this work, as detailed below, do not depend on this assumption. As the membranes are assumed to be fluid, their stretching is characterized by their area stretch moduli. F can then be reexpressed as

(2) $$\begin{array}{l} F=-TS+\frac{1}{2}\int \frac{{\left(\sigma_{xx}^{w}\right)}^{2}}{Y_{x}^{w}}+\frac{{\left(\sigma_{yy}^{w}\right)}^{2}}{Y_{y}^{w}}-\left(\frac{\nu_{xy}^{w}}{Y_{x}^{w}}+\frac{\nu_{yx}^{w}}{Y_{y}^{w}}\right)\\ \sigma_{xx}^{w}\sigma_{yy}^{w}dA^{w}+\underset{\alpha \in \left\{i,o\right\}}{\sum }\frac{K_{a}^{\alpha }}{2}\int {\left(u_{xx}^{\alpha }+u_{yy}^{\alpha }\right)}^{2}dA^{\alpha }. \end{array}$$

Here $\left(Y_{x}^{w},Y_{y}^{w},\nu_{xy}^{w},\nu_{yx}^{w}\right)$ are the two-dimensional Young's modulus and Poisson's ratio of the cell wall in the axial and circumferential (*x* and *y*) directions, $K_{a}^{\alpha }$ is the area stretch modulus, *u* denotes in-plane strains, σ denotes in-plane stresses, and *dA*^α^ denotes an area element. α represents an index: the inner and outer membranes are represented by α = *i* and α = *o*, respectively, while the cell wall is represented by α = *w*. Due to membrane fluidity, $u_{xx}^{\alpha }=u_{yy}^{\alpha }$ for α ∈ {*i, o*} when F is minimized (Wong and Amir, 2019). Furthermore, it is straightforward to show that $u_{xx}^{\alpha }$ does not vary with membrane position, so that the membrane stresses are isotropic and homogeneous (Wong and Amir, 2019).

For simplicity, we do not distinguish between the inner and outer membranes ($K=K_{a}^{i}=K_{a}^{o}$), so that all equations that follow hold for either membrane. We note here that the analogous free energy of a cell envelope comprising only a cytoplasmic membrane (indexed by *i*) can be written similarly to Equation (2). We find that it is equivalent to Equation (2) under the mapping *K* ↦ *K*/2 and σ^*i*^ + σ^*o*^ ↦ σ^*i*^. Hence, the case of a single membrane, as is relevant for Gram-negative cells without outer membranes or Gram-positive cells, can be readily accommodated by the considerations below.

To determine the stresses in the cellular envelope before cell wall defect formation, it suffices to determine $\sigma =\sigma_{xx}^{\alpha }=\sigma_{yy}^{\alpha }=2Ku_{xx}^{\alpha }=2Ku_{yy}^{\alpha }$ for α = *i, o*, from which the stresses in the cell wall follow by force balance. As shown previously (Wong and Amir, 2019), a direct application of force balance yields

(3) $$\begin{array}{l} \sigma \\ =\frac{K(-(A-2\pi \;r_{0}^{w}L_{0}^{w})Y_{x}^{w}Y_{y}^{w}+kTn_{s}(2Y_{x}^{w}(1-\nu_{yx}^{w})+Y_{y}^{w}(1-\nu_{xy}^{w}))}{2\pi \;r_{0}^{w}L_{0}^{w}(2K(Y_{x}^{w}(1-\nu_{yx}^{w})+Y_{y}^{w}(1-\nu_{xy}^{w}))+Y_{x}^{w}Y_{y}^{w})}. \end{array}$$

Here $A=\gamma \times 2\pi \;r_{0}^{w}L_{0}^{w}$ is the (inner or outer) membrane reference area, with γ denoting the reference membrane surface area ratio as compared to the cell wall, and $r_{0}^{w}$ and $L_{0}^{w}$ are the reference radius and length of the cell wall. Assuming the material parameters of the cellular envelope summarized in Supplementary Table 2, we solved Equation (3) to determine the stresses of the unbulged state, as shown in Supplementary Figure 2. We performed these calculations for (1) the physiological (wild-type) case; (2) a case in which the turgor was reduced ($n_{s}=4.7\times 10^{7}$); and (3) a case in which there is no outer membrane, as discussed above. These calculations were supported by numerically minimizing F, as previously described (Wong and Amir, 2019).

As the timescale of membrane lipid synthesis (tens of minutes; see Emiola et al., 2015) is anticipated to be longer than the timescale of bulging (seconds; see Figure 1D), we assume the membrane reference surface areas to remain unchanged, so that bulging corresponds to a quasi-equilibrium state in which the membrane reference surface areas limit bulge expansion. As shown previously (Wong and Amir, 2019), determining the equilibrium conformation of the cell envelope once a circular cell wall defect *A* of radius *r*_*d*_ is introduced amounts to solving a single, transcendental equation. In particular, the bulged conformation will exhibit a partially-subtended, spherical bulge (*B*; Supplementary Figure 2A) whose subtended angle, θ, is determined by the following *bulging equation*:

(4) $$\begin{array}{r} A=2\pi \;r_{0}^{w}L_{0}^{w}-\pi \;r_{d}^{2}\left(1-\frac{2}{1+\cos\theta }\right)\\ +\frac{3kTn_{s}}{4KY_{x}^{w}Y_{y}^{w}}\times \frac{\Phi \left(\theta \right)}{3{\left(r_{0}^{w}\right)}^{2}L_{0}^{w}\sin^{3}\theta +r_{d}^{3}\left(2+\cos\theta \right){\left(\cos\theta -1\right)}^{2}}, \end{array}$$

where $\Phi (\theta )=2r_{0}^{w}L_{0}^{w}(2Kr_{0}^{w}\sin^{3}\theta (2Y_{x}^{w}(1-\nu_{yx}^{w})+Y_{y}^{w}(1-\nu_{xy}^{w}))-r_{d}\sin^{2}\theta (Y_{x}^{w}Y_{y}^{w}+2K(Y_{x}^{w}(1-\nu_{yx}^{w})+Y_{y}^{w}(1-\nu_{xy}^{w})))-r_{d}^{3}Y_{x}^{w}Y_{y}^{w}\tan^{2}(\theta /2)\sin^{2}\theta$.

Assuming the material parameters of the cellular envelope summarized in Supplementary Table 2, we solved Equation (4) to determine the stresses and geometry of the bulged state, as shown in Supplementary Figure 2C, for (1) the physiological (wild-type) case; (2) a case in which the turgor was reduced; and (3) a case in which there is no outer membrane. These calculations were again supported by numerically minimizing F, as above. The bulge radius *R* was found by the relation *R* = *r*_*d*_ sin θ (Supplementary Figure 2A). Note that the model predicts bulging to occur, in principle, for any finite value of *r*_*d*_; nevertheless, for values much smaller than ~ 4–10 nm, the thickness of a lipid bilayer, the membrane cannot extrude through the cell wall defect and form a bulge. Accordingly, we anticipate the model to be physically accurate for large enough defect sizes, *r*_*d*_ ≳ 10 nm.

For the characteristic defect sizes considered here (*r*_*d*_ ~ 1 μm), the salient assumption of our model is that bulging corresponds to a quasi-equilibrium state in which force balance holds. We therefore sought to probe the implications of force balance on lysis dynamics. As we have assumed the inner and outer membranes to possess similar material properties, the model predicts the membranes to be similarly load-bearing and collectively resist stresses proportional to *pR*/2 (Supplementary Figure 2C) in the bulge. The strains collectively resisted are then *pR*/(4*K*). Assuming a fixed yield strain of the membranes then implies that, if the turgor pressure is reduced by a factor of two, then the yield bulge radius will be increased by a factor of two.

#### 4.10.2. Increased Membrane Tension After Bulging

Our model suggests that the increase in membrane tension after bulging is sufficient to trigger MSCs in many cells, as we explain next. According to the assumptions of force balance and identical membrane material properties in our model, the stress in each membrane at the time of lysis is *pR*_yield_/4, where *R*_yield_ is the yield bulge radius. For characteristic parameter values, our model also predicts physiological membrane stresses—that is, stresses in the unbulged state—of ~5 mN/m (Equation 3 and Supplementary Figure 2B). As the cellular volume remains essentially unchanged after bulging, increasing on average by <10% (Supplementary Figure 6A), the same turgor pressure and a typical value of 0.9 μm for *R*_yield_ (Figure 2E of the main text) predicts a mean membrane stress of ~11 mN/m at lysis. The right tail of the yield radii distribution (~10% of cells) corresponds to increased tensions of >20 mM/m, illustrating substantial variability between cells. For comparison, the MSCs MscS and MscL gate upon an increase in membrane tension in the range of 5–15 mN/m (Buda et al., 2016). The stress differential of >6 mN/m predicted by our model therefore suggests gating in a large fraction (~40%) of cells exhibiting yield bulge radii equal to, or greater than, 0.9 μm.

#### 4.10.3. Dependence of Bulge Size on Cell Width

As stated in the main text, we may consider a simple case of Equation (4) in which $Y_{x}^{w}=Y_{y}^{w}=Y$ and we neglect Poisson's effect ($\nu =\nu_{xy}^{w}=\nu_{yx}^{w}=0$). Accurate to the first order in *r*_*d*_/*R*, the solution of Equation (4) reduces to the following:

(5) $$\begin{array}{l} \theta \approx \frac{r_{d}}{R},\;R\approx \frac{6kTn_{s}Kr_{0}^{w}-4\left(\gamma -1\right)KY\pi {\left(r_{0}^{w}\right)}^{2}L_{0}^{w}}{kTn_{s}\left(4K+Y\right)}. \end{array}$$

For the parameters values of interest (Supplementary Table 2), the second term in the numerator of *R* in Equation (5) is dominated by the first; we therefore write

(6) $$\begin{array}{l} R\approx \frac{6kTn_{s}Kr_{0}^{w}}{kTn_{s}\left(4K+Y\right)}=\frac{6Kr_{0}^{w}}{4K+Y}. \end{array}$$

Thus, Equation (6) reveals a simple dependence of the bulge radius on the cell width: it predicts wider cells to yield larger bulges, with the subtended angle being determined by the ratio of cell wall hole radius to bulge radius. We note that wider *mreB*-titratable cells exhibit larger bulge radii, *R*, and similar values of subtended angles, θ, to thinner cells; this observation suggests larger values of the defect radius relative to thinner cells, consistent with the measurements shown in Supplementary Figure 8F.

### 4.11. Transport Model for Solute Outflow

#### 4.11.1. Change in Cellular Turgor and Volume Due to MSC Gating

To better understand the effect of MSCs on cellular turgor, we extended our biophysical model of cell envelope mechanics to predict the timescale of turgor loss due to MSC gating. A similar model, where the leakage of solutes arises from membrane defects, has been developed by some of us (Wong et al., 2021). As in Equation (1), we model the Gram-negative bacterial cell envelope as the combination of an elastic shell (the cell wall) sandwiched between two fluid membranes (the inner and outer membranes).

We assume that MSC gating is well described by the addition of nanoscale gaps of characteristic diameter ~3 nm in the membrane (Naismith and Booth, 2012; Buda et al., 2016). Thus, we model MSCs as holes with characteristic radius *r*_*c*_ ≈ 1.5 nm, which is smaller than the thickness of a lipid bilayer, ~4–10 nm. We next consider the electrochemical potential across the membrane, which comprises contributions due to the membrane potential and the chemical potential of cytoplasmic solutes. In *E. coli*, physiological estimates of the membrane potential are ~−100 mV (Ramos et al., 1976; Felle et al., 1980; Lo et al., 2007). In the case where the cellular turgor is predominantly generated by a concentration imbalance of an ion, the chemical potential, *E*, can be determined by the Nernst equation,

(7) $$\begin{array}{l} E=\frac{kT}{ze}\ln\left(\frac{C_{i}}{C_{o}}\right), \end{array}$$

where *e* is the elementary electric charge, *z* is the ion charge, and *C*_*i*_ and *C*_*o*_ are the concentrations of ions inside and outside the cell, respectively (Benarroch and Asally, 2020). We assume a typical cellular turgor pressure of ~0.5 atm (Supplementary Table 2) corresponding to a solute concentration difference of ~25 mM, and that K^+^ ions, for which *z* = 1, dominate the composition of all solutes in the cell, consistent with estimates of ion composition in *E. coli* (Milo and Phillips, 2015). We further assume an intracellular K^+^ concentration of ~25 mM, qualitatively consistent with typical estimates of 30–300 mM (Milo and Phillips, 2015). Equation (7) then implies a chemical potential of *E* ~ 140 mV, with larger predicted values for larger turgor pressures. As the membrane potential is smaller than, or comparable to, the predicted chemical potential and is also expected to collapse rapidly after channel gating due to inflow of Na^+^ and H^+^ (Lo et al., 2007; Booth, 2014), for simplicity, we consider the case in which solute outflow is driven predominantly by the chemical potential below.

For defects of characteristic diameter ~3 nm as assumed above, the hydrodynamic outflow of cytoplasmic contents—anticipated here to comprise mainly of water containing osmolytes—from inside to outside the cell is laminar and well-described by Poiseuille flow, so that the volumetric flow rate is

(8) $$\begin{array}{l} Q=\frac{\Delta PA^{2}}{8\pi \;\mu L_{c}}. \end{array}$$

Here Δ*P* is the pressure drop inside and outside the cell, $A=\pi \;r_{c}^{2}$ is the hole area, *L*_*c*_ is the hole length, and μ is the viscosity of the medium. Equation (8) is anticipated to be valid for describing the flow of osmolytes in water through high conductance, non-specific channels such as MscL and MscS, as reviewed previously (Haswell et al., 2011); we do not consider flow of ions through low conductance, selective ion channels here. We further note that, due to the entropic origin of turgor, *p* decreases with flow of solutes outside the cell and flow of water into the cell through the semi-permeable cell membranes. In turn, MSCs may stop gating due to there being less mechanical strain in the cell membranes.

For characteristic parameter values, as summarized in Supplementary Table 2, we find that, at the start of flow, *Q* ≈ 10^−21^ m^3^/s. Assuming this flow rate to be constant in time and a characteristic number of ~100 gated MSCs of all types in the cell—consistent with census estimates for MscL (Bialecka-Fornal et al., 2012; Chure et al., 2018)—a simple estimate shows that a flow comparable to the entire cellular volume out of the cell occurs on a timescale of ~100 s, a timescale comparable to the mean bulge lifetimes observed in our lysis experiments.

We now perform a more detailed analysis, taking into account the decrease of turgor and cell volume with flow of solutes outside the cell. A characteristic value of the diffusion constant of ions in water is *D* ≈ 10^−9^ m^2^/s (Cussler, 1997), so that a typical root-mean-square distance traveled by an ion per second is 10 μm. Accordingly, we assume solutes to be significantly diluted once outside the cell, so that Δ*P* = *p*, the turgor pressure of the cell. Viewing *n*_*s*_, *p*, *Q*, and the cell volume, *V*, as time-dependent quantities that change with flow of solutes out of the cell, we therefore write:

(9) $$\begin{array}{l} Q\left(t\right)=\frac{\pi \;p\left(t\right)r_{c}^{4}}{8\mu L_{c}},\;p\left(t\right)=\frac{kTn_{s}\left(t\right)}{V\left(t\right)},\;\frac{dn_{s}\left(t\right)}{dt}=-\frac{NQ\left(t\right)n_{s}\left(t\right)}{V\left(t\right)}, \end{array}$$

where N is the number of gated MSCs. We note here that *r*_*c*_ is assumed to be constant over time, in contrast to our previous model examining membrane defects (Wong et al., 2021). It therefore remains to determine *V*(*t*). As mentioned in the main text, we will first consider the analytically tractable case of a cell with no bulges, then verify that the theoretical predictions are quantitatively similar in the case of a cell with a bulge.

#### 4.11.2. Elastic Determination of the Cellular Volume

Due to the possibility of water flow into the membrane as the number of solutes are modulated, we hypothesize the cellular volume to be determined by the equilibration of the elastic strain energies in Equation (1). In particular, given the turgor pressure, *p*(*t*), the cell envelope is free to change its dimensions to minimize the free energy. This timescale separation is supported by the following estimate. The bulk flow of water through the cell membranes is described by

(10) $$\begin{array}{l} \frac{dV_{\text{water}}}{dt}=L_{p}A_{\text{cell}}p, \end{array}$$

where *L*_*p*_ is the hydraulic conductivity of the membranes and *A*_cell_ is the total membrane surface area (Sperelakis, 1995). For characteristic values of these parameters, as summarized in Supplementary Table 2, we find that a typical ~40% change in cellular volume occurs within ~1 s. Hence, for the timescale of interest (~1 s) here, we find that water flow should indeed occur fast enough for the cell to be in equilibrium.

We therefore determine *V*(*t*) by finding the elastic stresses in the equilibrium conformation. For this, we resort to a linear theory and assume, as above, a linear-elastic, isotropic cell wall, with reference radius and lengths $r_{0}^{w}$ and $L_{0}^{w}$, respectively, and (two-dimensional) Young's modulus and Poisson's ratio *Y*^*w*^ and ν^*w*^, respectively. Moreover, as before, we view the two membranes as materially identical and fluid in-plane, so that their stretching is governed by their area-stretch modulus, *K* = *K*^*i*^ = *K*^*o*^, and reference surface area ratio, $\gamma =A_{0}^{i}/A_{0}^{w}=A_{0}^{o}/A_{0}^{w}$, where $A_{0}^{w}$ is the reference cell wall surface area, and $A_{0}^{i}$ and $A_{0}^{o}$ are the inner and outer membrane reference surface areas, respectively (Wong and Amir, 2019). The free energy of Equation (1) can then be expressed as

(11) $$\begin{array}{r} F=\frac{1}{2Y^{w}}\int \left[{\left(\sigma_{xx}^{w}\right)}^{2}+{\left(\sigma_{yy}^{w}\right)}^{2}-2\nu^{w}\sigma_{xx}^{w}\sigma_{yy}^{w}\right]dA^{w}\\ +2K\int {\left(u^{i}\right)}^{2}dA^{i}+2K\int {\left(u^{o}\right)}^{2}dA^{o}-TS, \end{array}$$

where the integrals are over the deformed surface areas, $\sigma_{xx}^{w}$ and $\sigma_{yy}^{w}$ are cell wall stresses, and *u*^*i*^ and *u*^*o*^ are inner and outer membrane strains, respectively. As detailed in the previous section (see section 4.10), the form of $E_{\text{strain}}^{i}$ and $E_{\text{strain}}^{o}$ in Equation (11) arises from the fluid in-plane nature of the membranes; it follows from this that the membrane strains and stresses are isotropic and spatially homogeneous (Wong and Amir, 2019). As the cell wall is cylindrical, its strains and stresses will also be spatially homogeneous, but not necessarily isotropic.

Depending on the values of γ, *K*, and *p*, we note that, in general, the deformed membrane dimensions can be different from each other and those of the cell wall: in the limit of small 0 < γ ≪ 1 and *p*/*K* ≪ 1, for instance, the free energy is minimal when the inner membrane forms a spherical vesicle inside the cell and the cell wall and outer membrane bear no load. However, we may anticipate a parameter regime in which all envelope layers bear some load (below). Then, by symmetry of the inner and outer membranes, *u* = *u*^*i*^ = *u*^*o*^ and the membrane stresses σ = σ^*i*^ = σ^*o*^; moreover, these quantities will all be nonzero. As detailed in the previous section, the mechanical stresses will be related to the strains by the following constitutive relations (Wong and Amir, 2019):

(12) $$\begin{array}{r} \sigma_{xx}^{w}=\frac{Y^{w}}{1-{\left(\nu^{w}\right)}^{2}}\left(u_{xx}^{w}+\nu^{w}u_{yy}^{w}\right),\\ \sigma_{yy}^{w}=\frac{Y^{w}}{1-{\left(\nu^{w}\right)}^{2}}\left(u_{yy}^{w}+\nu^{w}u_{xx}^{w}\right),\;\sigma =2Ku. \end{array}$$

Here, the cell wall strains $u_{xx}^{w}$ and $u_{yy}^{w}$ correspond to the stresses $\sigma_{xx}^{w}$ and $\sigma_{yy}^{w}$. Furthermore, the linear strain-displacement relations are

(13) $$\begin{array}{r} u_{xx}^{w}=\frac{r-r_{0}^{w}}{r_{0}^{w}},\;u_{yy}^{w}=\frac{L-L_{0}^{w}}{L_{0}^{w}},\\ u=\frac{A^{i}-A_{0}^{i}}{2A_{0}^{i}}=\frac{A^{o}-A_{0}^{o}}{2A_{0}^{o}}, \end{array}$$

where *r* and *L* are the deformed cell wall radius and length, respectively. Assuming that the membranes share the same deformed radius and length, we substitute Equations (12) and (13), as well as the relation $n_{w}=\pi \;r^{2}L/m_{w}$, where *m*_*w*_ is the volume occupied per water molecule, into Equation (11). From this, we find that F can be rewritten as a function of two unknowns, *r* and *L*, and several parameters including the elastic constants, γ, and *n*_*s*_. Hence, we will minimize F over *r* and *L*, from we determine all associated elastic quantities.

As mentioned above, we anticipate that, for typical cells, the membrane reference areas will be similar to that of the cell wall, so that |γ − 1| ≪ 1 (Wong and Amir, 2019). Furthermore, we anticipate all cell envelope layers to be load-bearing and in contact in the deformed state, so that we may suppose a common value of the deformed cell length and radius among all envelope layers; these may be expressed as $L=L_{0}^{w}+\delta L$ and $r=r_{0}^{w}+\delta r$, where δ*L* and δ*r* are viewed as small relative to $L_{0}^{w}$ and $r_{0}^{w}$, respectively. We note that the general case in which this assumption is not satisfied involves a minimization of the free energy over additional variables describing the membrane geometries (Wong et al., 2017), which makes deriving analytic expressions for $\sigma_{xx}^{w}$ and $\sigma_{yy}^{w}$ more complicated than presented here. Next, we make the following small-variable assumptions: *n*_*s*_/*n*_*w*_≪1 and δ*r*/*r*, δ*L*/*L* = *O*(ε), where ε≪1, consistent with the linear theory. In particular, since characteristic parameter values give $n_{s}/n_{w}\approx 10^{-4}$ and we may expect |γ − 1| ≈ 0.01 and $u,u_{xx}^{w},u_{yy}^{w}\approx 0.01$ (Supplementary Table 2), we will expand F to first order in *n*_*s*_ about 0, second order in ε about 0, and second order in γ about 1. Doing so, and analytically solving for the values of δ*L* and δ*r* which minimize F, upon substitution of the solution into Equations (12) and (13) we find

(14) $$\begin{array}{l} \sigma_{xx}^{w}=\frac{\left(\gamma -1\right)KY^{w}}{Y+2K\left(1-\nu^{w}\right)}+\frac{kTn_{s}\left(K-K\nu^{w}+2Y^{w}\right)}{2\pi \;r_{0}^{w}L_{0}^{w}\left[2K\left(1-\nu^{w}\right)+Y^{w}\right]}\\ \;+\;O\left(\varepsilon^{2}\right)+O\left[{\left(\frac{n_{s}}{n_{w}}\right)}^{2}\right]+O\left({\left(\gamma -1\right)}^{2}\right)\\ \;+\;O\left(\left(\gamma -1\right)\varepsilon \right)+O\left(\frac{\left(\gamma -1\right)n_{s}}{n_{w}}\right)+O\left(\frac{\varepsilon n_{s}}{n_{w}}\right). \end{array}$$

Accurate to the same order, we have

(15) $$\begin{array}{r} \sigma_{yy}^{w}=\frac{\left(\gamma -1\right)KY^{w}}{Y^{w}+2K\left(1-\nu^{w}\right)}+\frac{kTn_{s}\left(K-K{\left(\nu^{w}\right)}^{2}+Y^{w}\right)}{2\pi \;r_{0}^{w}L_{0}^{w}\left[2K\left(1-\nu^{w}\right)+Y^{w}\right]},\\ \sigma =\frac{K\left[3kTn_{s}\left(1-\nu^{w}\right)-2\pi \;r_{0}^{w}L_{0}^{w}\left(\gamma -1\right)Y^{w}\right]}{4\pi \;r_{0}^{w}L_{0}^{w}\left[2K\left(1-\nu^{w}\right)+Y^{w}\right]}. \end{array}$$

It is straightforward to verify that $\sigma_{xx}^{w}+2\sigma =\frac{kTn_{s}}{\pi r_{0}^{w}L_{0}^{w}}$ and $\sigma_{yy}^{w}+2\sigma =\frac{kTn_{s}}{2\pi \;r_{0}^{w}L_{0}^{w}}$, so that Laplace's law (Wong and Amir, 2019) is satisfied. We note here that the stresses of Equations (14) and (15) do not vanish when *n*_*s*_ = 0, due to the simplifying assumption of a common value of the deformed cell length and radius among all envelope layers. When *n*_*s*_ = 0, the membranes are free to assume dimensions that minimize their stretching energies and, in general, the assumption of a common value of the deformed cell length and radius among all envelope layers no longer holds. However, for characteristic values of *n*_*s*_ relevant to *E. coli*, as considered in this work, we have previously shown that this assumption is valid (Wong and Amir, 2019). This assumption then results in the simplified expressions for the stresses given by Equations (14) and (15).

Finally, by viewing the stresses in Equations (14) and (15) as functions of time through their dependence on *n*_*s*_ = *n*_*s*_(*t*) and finding the corresponding time-dependent strains through the linear constitutive relations of Equation (12), we can write a closed-form expression for *V*(*t*) as:

(16) $$\begin{array}{l} V\left(t\right)=\pi \;{\left(r_{0}^{w}\right)}^{2}L_{0}^{w}\left[1+2u_{xx}^{w}\left(t\right)+u_{yy}^{w}\left(t\right)\right]. \end{array}$$

Henceforth, all equalities will be accurate to the orders shown in Equations (14).

#### 4.11.3. The Dynamical Equation

Iteratively substituting Equations (12)–(16) into Equation (9), we find that a single equation governs the dynamics of solute flow which, in turn, determines all other quantities:

(17) $$\begin{array}{l} \frac{dn_{s}\left(t\right)}{dt}=-\frac{\pi NkTn_{s}^{2}\left(t\right)r_{c}^{4}{\left(Y^{w}\right)}^{2}{\left(Y^{w}+2K\left(1-\nu^{w}\right)\right)}^{2}}{2\mu L_{d}{\left(r_{0}^{w}\right)}^{2}{\left[2\pi \;r_{0}^{w}L_{0}^{w}Y^{w}\left(Y^{w}+K\left(1-\nu^{w}\right)\left(3\gamma -1\right)\right)+kTn_{s}\left(t\right)\left(K-K{\left(\nu^{w}\right)}^{2}+Y^{w}\left(5-4\nu^{w}\right)\right)\right]}^{2}}, \end{array}$$

where $n_{s}\left(t=0\right)=n_{s}^{0}$, the initial number of solutes inside the cell. We numerically solved this equation for the parameter values of interest (Supplementary Table 2).

#### 4.11.4. Timescales of Solute Flow and Increases in Bulge Stress

Solving the dynamical Equation (17) numerically for the parameter values summarized in Supplementary Table 2, we find that the model predicts a gradual decrease in turgor on the timescale of ~100 s, followed by accompanying decreases in volume (Supplementary Figure 3). Importantly, we find that this decrease in turgor is insufficient to overcome the increase in membrane stresses due to bulge growth: based on our experimental observations that the mean bulge radius is ~1 μm after ~200 s (Figures 1, 2 of the main text), we find that a typical bulge radius growth rate of 0.005 μm/s is large enough so that the corresponding estimate of bulge stress, σ = *p*(*t*)*R*(*t*)/2, is monotonically increasing in time (Supplementary Figure 3), where *p*(*t*) is determined by the solution of Equation (17) and *R*(*t*) is taken to be *R*(*t*) = 0.005 μm/s × *t* as a phenomenological approximation to our observations. We note here that heterogeneous dynamics in *R*(*t*), as would be expected if the bulge does not grow constantly in time, would lead to different predicted behaviors of σ.

While we have modeled cells without bulges, we note that considering the bulge geometry would make an expression of the form Equation (17) intractable to obtain, as the transcendental bulging equation (Equation 4) would need to be solved to obtain the stresses of Equations (14) and (15). Nevertheless, we have previously shown that numerical solutions of the bulging equation indicate the cell volume of a bulged cell to be approximately equal to that on an unbulged cell (Wong and Amir, 2019). As the model predictions are robust to variations in *V*(*t*) (Supplementary Figure 3), we expect that the inclusion of a bulge to the analysis has a limited effect on the model predictions. In confirmation of this, we numerically solved Equation (9) in the case where *V*(*t*) is determined by the bulging equation, and observed that the model predictions were, as expected, quantitatively similar to the unbulged case (Supplementary Figure 4A).

In conclusion, our modeling results demonstrate how MSCs may fail to resist lysis. Furthermore, these results suggest that, while solute outflow may decrease turgor in cells, the turgor decrease is counteracted by bulge growth, leading to increasing mechanical stresses until the cell lyses.

#### 4.11.5. Comparison With Cells Under Hypoosmotic Shock

We summarize the main differences between our model and that previously developed for cells under hypoosmotic shock (Buda et al., 2016). In both cases, the model predicts the outflow of solutes in response to the gating of MSCs; for bulged cells, however, our model suggests that the outflow of solutes is insufficient to decrease the mechanical stresses in the bulge, since decreases in the turgor pressure (*p*) are compensated for by increases in the bulge radius (*R*) such that the mechanical stresses in the bulge (equal to *pR*/2) increases in time (Supplementary Figure 3). In contrast, in experiments involving hypoosmotic shocks, cells do not exhibit bulges and the mechanical stresses in the cell envelope are proportional to *pr*, where *r* is the cell radius. As *r* does not significantly increase in time and *p* decreases with solute outflow, the mechanical stresses in the cell envelope decrease, instead of increase, in time. This difference is further illustrated in Supplementary Figure 4B, in which we plot the bulge areal strain, defined as the quantity $2u=\left(A^{i}-A_{0}^{i}\right)/A_{0}^{i}=\left(A^{o}-A_{0}^{o}\right)/A_{0}^{o}=p\left(t\right)R\left(t\right)/4K$, after MSC gating; note that a factor of 2 enters in the denominator due to the presence of two membranes. For comparison, the membrane areal strain of a cylindrical cell after MSC gating, but without bulging, is also plotted in Supplementary Figure 4B.

### 4.12. Generality of Model Assumptions: Osmotic Nature of the Periplasm

In our model, we have assumed that the turgor pressure is generated by solutes in the cytoplasm (Hussain et al., 2018; Wong and Amir, 2019; Wong et al., 2021) and exerts a force on all three layers of the Gram-negative bacterial cell envelope. This assumption is consistent with viewing the periplasm as an effectively rigid body. We have previously suggested (Hussain et al., 2018) that a case in which the cytoplasm is isoosmotic with the periplasm (Sochacki et al., 2011; Erickson, 2017, 2021), such that the OM is effectively rigid and the only load-bearing layer of the cellular envelope, is inconsistent with the mechanical stability of the periplasm because the bending energies of the membranes in Equation (1) are smaller for cylindrical shapes of larger radius: thus, in rod-like cells with sufficient IM surface area, the IM is predicted to press against the cell wall and OM and squeeze out any isoosmotic periplasmic space (see Hussain et al., 2018 for a detailed discussion). Nevertheless, this case can be accommodated in our model by (1) setting *K* = 0 and the elastic moduli of the cell wall, *Y*^*w*^, to be that of the rigid OM in the cylindrical bulk of the cell, and (2) viewing *K* as the area-stretch modulus of the cell wall-decoupled, fluid OM in the bulge. As a previous study has indicated the presence of cytoplasm in membrane bulges (Yao et al., 2012), our model suggests that the formation of a membrane bulge after β-lactam treatment depends on the untethering of proteins anchoring the IM and OM to the cell wall: such proteins may include transmembrane cell wall synthases including the Rod complex, Braun's lipoprotein, and OmpA (Movva et al., 1980; Silhavy et al., 2010; van Teeffelen and Renner, 2018).

We note that, in both cases, the bulge stresses are identical and equal to σ = *p*(*t*)*R*(*t*)/2. This observation indicates that the prediction of turgor pressure as a driver of lysis applies to both cases. Furthermore, in the case of an isoosmotic periplasm, the dynamical equation for solute outflow out of a rod-like cell, corresponding to Equation (17), is

(18) $$\begin{array}{l} \frac{dn_{s}\left(t\right)}{dt}=-\frac{\pi NkTr_{c}^{4}{\left(Y^{w}\right)}^{4}n_{s}{\left(t\right)}^{2}}{2\mu L_{d}{\left(r_{0}^{w}\right)}^{2}{\left[2\pi \;r_{0}^{w}L_{0}^{w}{\left(Y^{w}\right)}^{2}+kTn_{s}\left(t\right)Y^{w}\left(5-4\nu^{w}\right)\right]}^{2}}, \end{array}$$

where $r_{0}^{w},L_{0}^{w},Y^{w}$, and ν^*w*^ describe quantities relevant to the effectively rigid outer membrane. For characteristic parameter values relevant to *E. coli* (Supplementary Table 2), the predictions of Equation (18) are quantitatively similar to those of Equation (17). Thus, we anticipate that the model predictions for the effects of hyperosmotic shock and MSC gating on lysis are qualitatively similar between both cases. We anticipate that further studies will better discriminate between these two sets of model assumptions by clarifying the osmotic nature of the periplasm.

## Data Availability Statement

The original contributions presented in the study are included in the article/Supplementary Material, further inquiries can be directed to the corresponding author/s.

## Author Contributions

FW, AA, and LR conceived the project. FW, SW, CL, TP, EG, AA, and LR designed the research. FW, SW, RH, SH, OA, HZ, and LR performed the research. All authors analyzed the data. FW, SW, TP, EG, AA, and LR wrote the paper with the assistance of all authors.

## Conflict of Interest

The authors declare that the research was conducted in the absence of any commercial or financial relationships that could be construed as a potential conflict of interest.

## Publisher's Note

All claims expressed in this article are solely those of the authors and do not necessarily represent those of their affiliated organizations, or those of the publisher, the editors and the reviewers. Any product that may be evaluated in this article, or claim that may be made by its manufacturer, is not guaranteed or endorsed by the publisher.
