# Supplementary material for: Understanding Beta-Lactam-Induced Lysis at the Single-Cell Level
Source: Front Microbiol. 2021 Jul 27;12:712007. doi: 10.3389/fmicb.2021.712007 (PMC8372035; doi:10.3389/fmicb.2021.712007)
Supplement: Supplementary file 7 [file Data_Sheet_1.pdf]

Supplementary Material

SUPPLEMENTARY MOVIES

Movie S1: **Lysis dynamics of a population of *E. coli* cells.** Movie S1 shows a representative population of wild-type *E. coli* (strain MG1655) cells in the time after cephalexin treatment. The time is indicated in hours:minutes: seconds, the time between frames is 1 min, and the timelapse covers a period of about 1.5 hrs. The field of view is approximately 126 μm $\times$ 128 μm.

Movie S2: **Lysis dynamics of a representative *E. coli* cell.** Movie S2 shows a representative wild-type *E. coli* (strain MG1655) cell in the time after cephalexin treatment. The time is indicated in minutes:seconds, the time between frames is 0.1 s, and the timelapse covers a period of about 15 mins. The field of view is approximately 16 μm $\times$ 13 μm.

Movie S3: **Lysis dynamics of a representative, osmotically-stabilized *E. coli* cell.** Movie S3 shows a representative wild-type *E. coli* (strain MG1655) cell in the time after cephalexin treatment, stabilized by flow of hyperosmotic media (250 mM sorbitol). The time is indicated in hours:minutes:seconds, the time between frames is 1 s, and the timelapse covers a period of about 1.25 hrs. The field of view is approximately 13 μm $\times$ 13 μm.

Movie S4: **Lysis dynamics of a population of Δ*aqpZ* *E. coli* cells.** Movie S4 shows a representative population of Δ*aqpZ* *E. coli* cells in the time after cephalexin treatment. The time is indicated in hours:minutes:seconds, the time between frames is 1 min, and the timelapse covers a period of about 1.5 hrs. The field of view is approximately 121 μm $\times$ 129 μm.

Movie S5: **Lysis dynamics of a population of *E. coli* MSC mutants.** Movie S5 shows a representative population of Δ7 *E. coli* cells in the time after cephalexin treatment. The time is indicated in minutes:seconds, the time between frames is 1 min, and the timelapse covers a period of about 45 mins. The field of view is approximately 127 μm $\times$ 94 μm.

Movie S6: **Lysis dynamics of a population of *mreB*-titratable *E. coli* cells.** Movie S6 shows a representative population of *mreB*-titratable *E. coli* cells in the time after cephalexin treatment, at a concentration of 3 ng/mL aTc. The time is indicated in hours:minutes:seconds, the time between frames is 1 min, and the timelapse covers a period of about 2 hrs. The field of view is approximately 128 μm $\times$ 130 μm.

SUPPLEMENTARY REFERENCES

Amir, A., Babaeipour, F., McIntosh, D. B., Nelson, D. R., and Jun, S. (2014). Bending forces plastically deform growing bacterial cell walls. *Proc. Natl. Acad. Sci. USA* 111, 5778–5783

Bialecka-Fornal, M., Lee, H. J., DeBerg, H. A., Gandhi, C. S., and Phillips, R. (2012). Single-cell census of mechanosensitive channels in living bacteria. *PLoS ONE* 7, 0033077

Buda, R., Liu, Y., Yang, J., Hegde, S., Stevenson, K., Bai, F., et al. (2016). Dynamics of *Escherichia coli*'s passive response to a sudden decrease in external osmolarity. *Proc. Natl. Acad. Sci. USA* 113, E5838–E5846

Cayley, D. S., Guttman, H. J., and Record, M. T. (2000). Biophysical characterization of changes in amounts and activity of *Escherichia coli* cell and compartment water and turgor pressure in response to osmotic stress. *Biophys. J.* 78, 1748–1764

Çetiner, U., Rowe, I., Schams, A., Mayhew, C., Rubin, D., Anishkin, A., et al. (2017). Tension-activated channels in the mechanism of osmotic fitness in *Pseudomonas aeruginosa*. *J. Gen. Physiol.* 149, 595–609

Chure, G., Lee, H. J., Rasmussen, A., and Phillips, R. (2018). Connecting the dots between mechanosensitive channel abundance, osmotic shock, and survival at single-cell resolution. *J. Bacteriol.* 200, e00460–18

Deng, Y., Sun, M., and Shaevitz, J. W. (2011). Direct measurement of cell wall stress stiffening and turgor pressure in live bacterial cells. *Phys. Rev. Lett.* 107, 158101

Hegde, S. (2020). Dissertation: *Escherichia coli*'s response to hypoosmotic shocks. *Univ. Edinburgh*

Lan, G., Wolgemuth, C. W., and Sun, S. X. (2007). Z-ring force and cell shape during division in rod-like bacteria. *Proc. Natl. Acad. Sci. USA* 104, 16110–16115

Naismith, J. H. and Booth, I. R. (2012). Bacterial mechanosensitive channels—MscS: Evolution's solution to creating sensitivity in function. *Annu. Rev. Biophys.* 41, 157–177

Phillips, R., Kondev, J., Garcia, H., and Theriot, J. (2012). *Physical Biology of the Cell* (Taylor and Francis)

Sperelakis, N. (1995). *Cell Physiology Source Book: Essentials of Membrane Biophysics* (Academic Press)

Sun, Y., Sun, T.-L., and Huang, H. W. (2014). Physical properties of *Escherichia coli* spheroplast membranes. *Biophys. J.* 107, 2082–2090

Tuson, H. H., Auer, G. K., Renner, L. D., Hasebe, M., Tropini, C., Salick, M., et al. (2012). Measuring the stiffness of bacterial cells from growth rates in hydrogels of tunable elasticity. *Mol. Microbiol.* 85, 874–891

Wong, F. and Amir, A. (2019). Mechanics and dynamics of bacterial cell lysis. *Biophys. J.* 116, 2378–2389

Yao, X., Jericho, M., Pink, D., and Beveridge, T. (1999). Thickness and elasticity of Gram-negative murein sacculi measured by atomic force microscopy. *J. Bacteriol.* 181, 6865–6875

Yao, Z., Kahne, D., and Kishony, R. (2012). Distinct single-cell morphological dynamics under beta-lactam antibiotics. *Mol. Cell* 48, 705–712

Zheng, H., Ho, P.-Y., Jiang, M., Tang, B., Liu, W., Li, D., et al. (2016). Interrogating the *Escherichia coli* cell cycle by cell dimension perturbations. *Proc. Natl. Acad. Sci. USA* 113, 15000–15005

SUPPLEMENTARY FIGURES

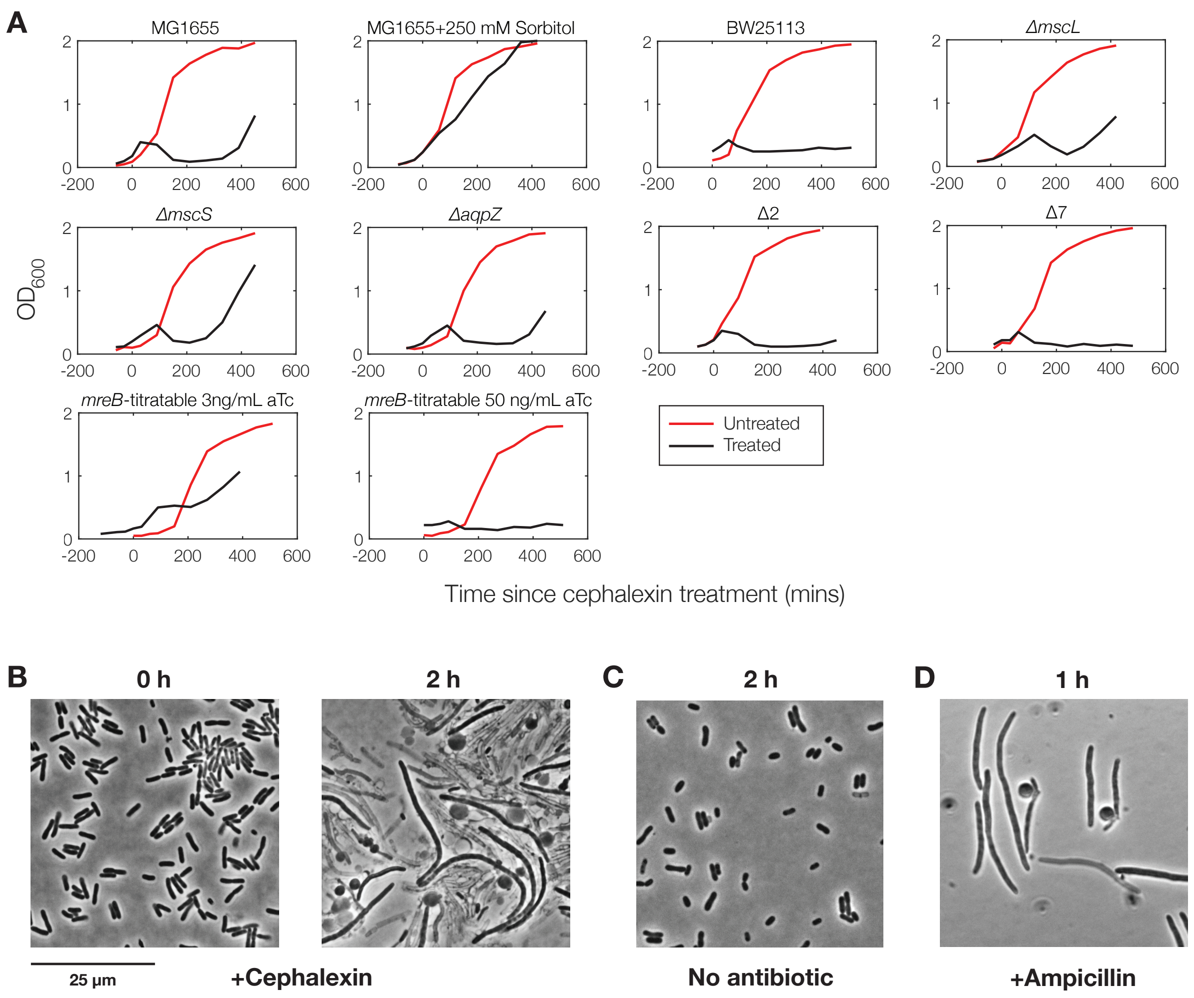

Figure S1. Experiments examining cell growth and responses to antibiotic treatment.
(A) OD_{600} measurements of *E. coli* cultures untreated or treated by cephalexin across various strains and conditions. Each plot shows a different strain or condition considered in this study, and cephalexin (50 μg/mL) was added to treated cultures in early-log phase, corresponding to an OD_{600} of approximately 0.2. Cells were grown in the conditions indicated before cephalexin treatment, so that the appropriate phenotypes were induced, with the exception of osmotic shocks, for which 250 mM sorbitol was added at the same time as cephalexin. Each curve is representative of two biological replicates.
(B) Phase-contrast image of a population of *E. coli* (strain MG1655) cells immediately after cephalexin (50 μg/mL) treatment (left), and phase-contrast image of the same population treated with cephalexin for 2 hrs (right), as shown in Figure 1B and 1C in the main text.
(C) Phase-contrast image of a control population of *E. coli* cells corresponding to (B), but without cephalexin treatment, after 2 h.
(D) Phase-contrast image of a population of *E. coli* cells treated with ampicillin (50 μg/mL) for 1 hr.

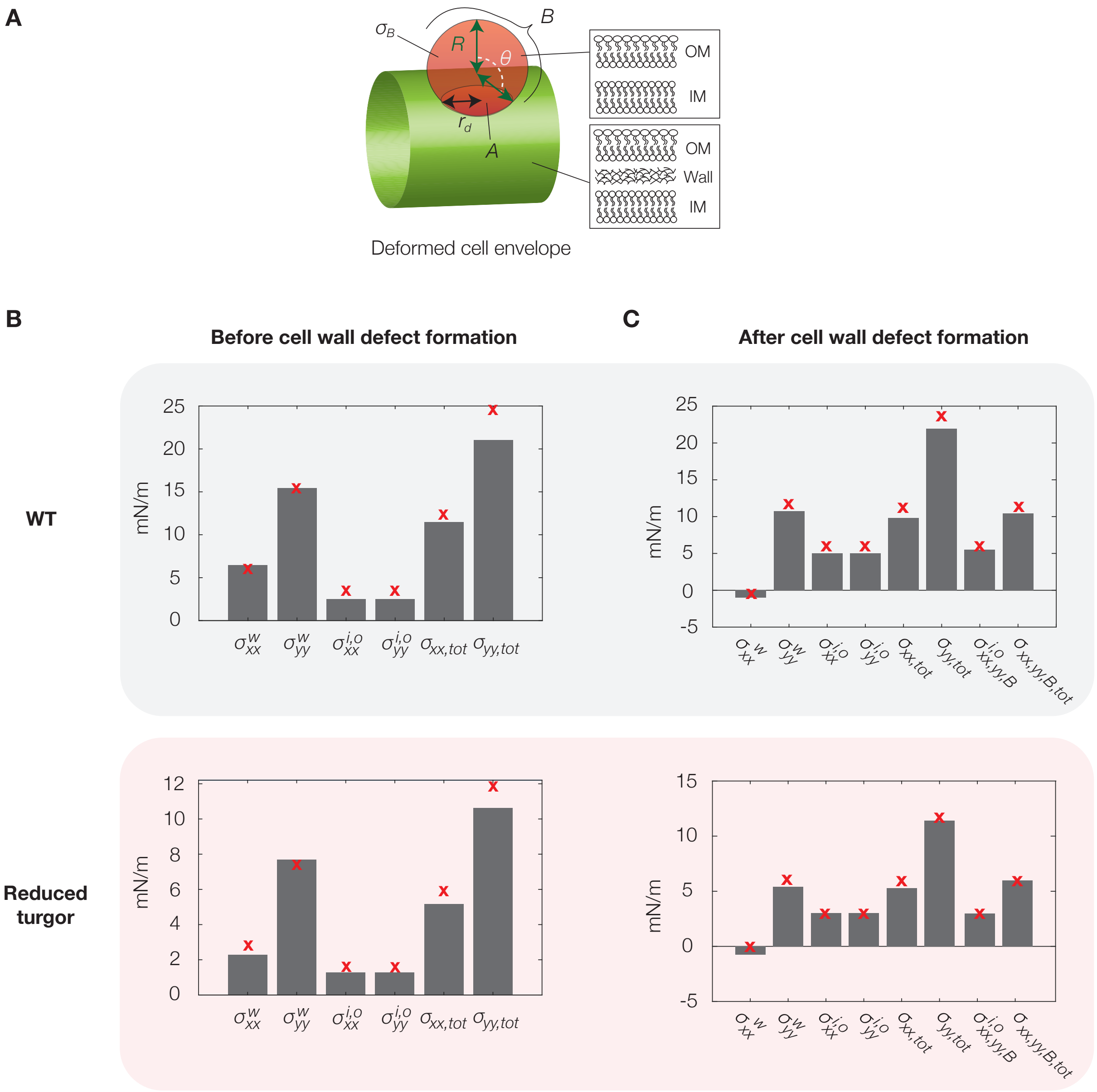

Figure S2. Model predictions for different perturbations considered in this work.
(A) A schematic of the model, showing a bulged cell and its different variables. Here R denotes the radius of the bulge (indicated in the figure by B), θ the angle subtended by the bulge, r_d the radius of the cell wall defect (indicated in the figure by A), and σ_B the elastic stress in the bulge. IM and OM, inner membrane and outer membrane. The schematic is reproduced from (Wong and Amir, 2019).
(B) Predicted cell envelope stress profiles before cell wall defect formation, for *E. coli* cells across varying conditions. The model parameters are summarized in Table S2. The '$\times$' symbols indicate analytical predictions, while the bars indicate numerical results. "Reduced turgor" refers to the case where the number of solutes contained in the cell, corresponding to the turgor pressure, is halved. The subscript *tot* refers to the total stresses.
(C) Same as (B), but after cell wall defect formation. In all cases, a constant cell wall defect radius of $r_d = 0.5\ \mu$m is assumed. The subscript B refers to stresses in the bulge.

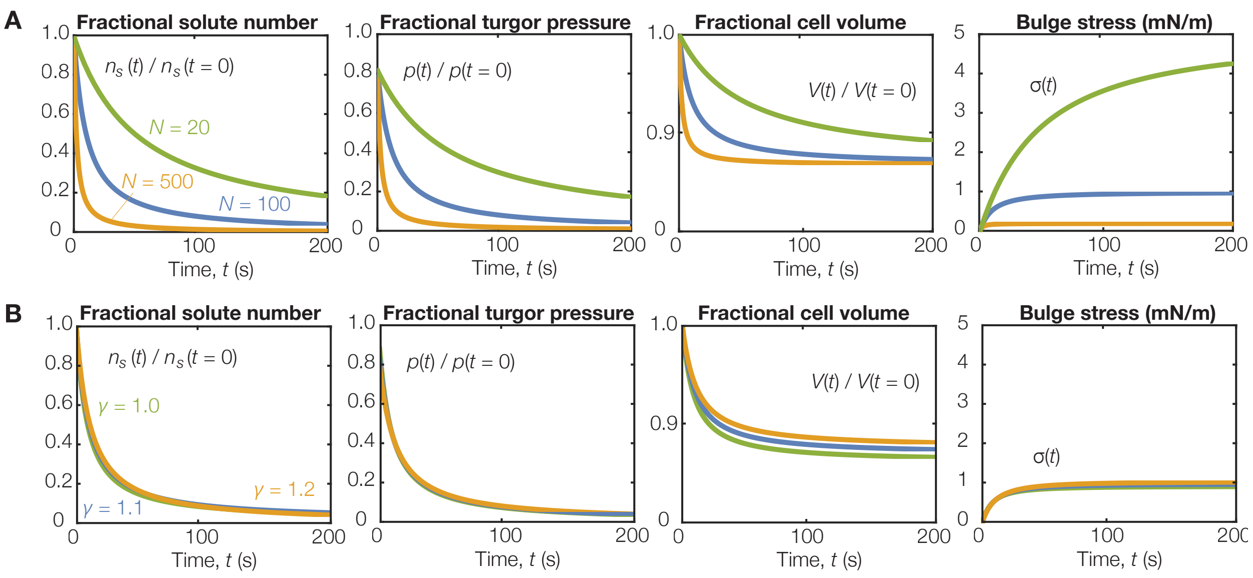

Figure S3. Transport model predictions for cellular variables after MSC gating. Plots of solute number, turgor pressure, cell volume, and bulge stress against time after MSC gating, for the parameter values summarized in Table S2. Model predictions describe the response of these variables to MSC gating and were determined by solving Eq. (17) in the *Methods*. Sensitivity analyses to (A) the number of gated MSCs, $\mathcal{N}$, with $\gamma = 1.1$ and (B) the reference membrane area ratio, γ, with $\mathcal{N} = 100$, are shown.

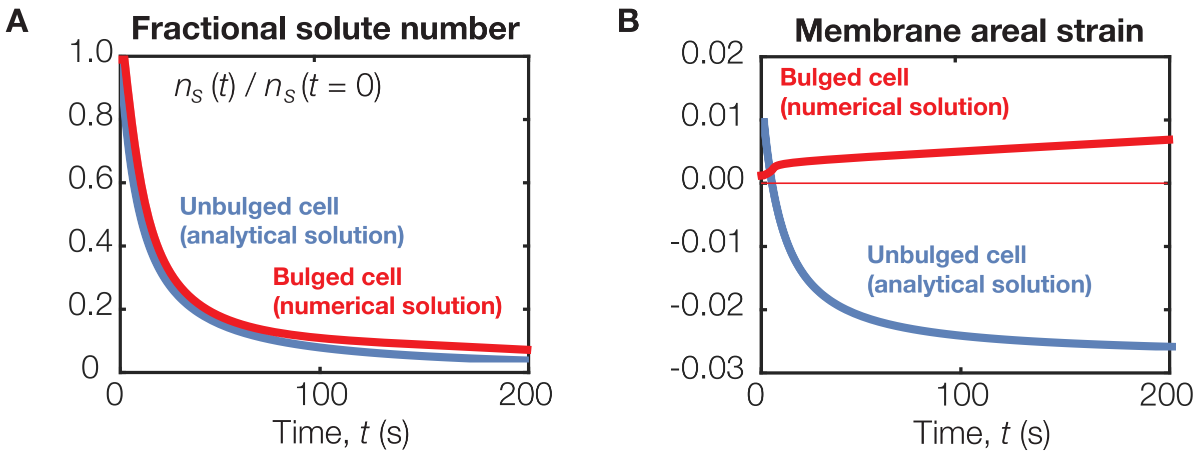

Figure S4. The bulged geometry does not change the predictions of solute outflow, but leads to increasing membrane stresses, in contrast to hypoosmotic shocks.
(A) Shown is a plot of the predicted solute number as a function of time, corresponding to Figure S3 and the parameter values for *E. coli* summarized in Table S2, with $\mathcal{N} = 100$ and $\gamma = 1.1$, for an unbulged cell (blue curve) as compared to a bulged cell (red curve). The numerical prediction for the unbulged cell is found by solving Eq. (17) in the *Methods*. The numerical prediction for the bulged cell is found by solving Eq. (9) in the *Methods*, assuming that the cell volume is determined by the bulging equation, Eq. (4) in the *Methods*.
(B) A plot of the bulge membrane strain as a function of time (red curve) and a plot of the membrane areal strain in a cylindrical cell without a bulge (blue curve). The numerical prediction for the bulge membrane strain is found by solving Eqs. (4) and (9) in the *Methods*. The numerical prediction for the membrane areal strain in a cylindrical cell is found by solving Eq. (17) in the *Methods*. In both cases, the parameters values used are summarized in Table S2, with $\mathcal{N} = 100$ and $\gamma = 1.1$. MSCs are assumed to be gated at $t = 0$.

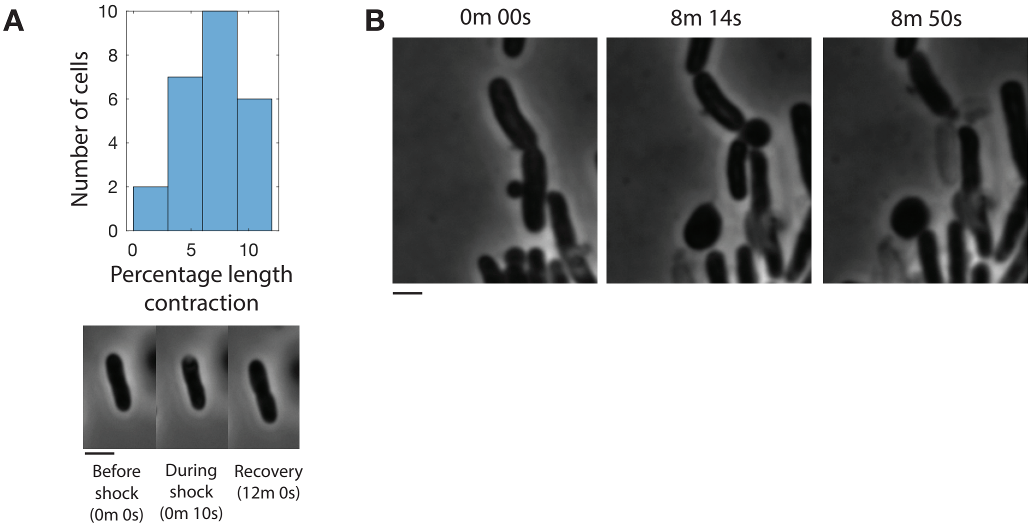

Figure S5. Control osmotic shock experiments and observation of membrane bulge detachment. (A) Hyperosmotic shock measurements in control, untreated *E. coli* (strain MG1655) cells. The distribution of percentage changes in cell length after hyperosmotic shock by flow of growth media containing 500 mM sorbitol is shown for 25 cells. The inset shows a phase-contrast timelapse of one cell, with time indicated in minutes (m) and seconds (s). Scale bar, 1 μm.
(B) A sequence of timelapse images wherein a membrane bulge detaches from a bulged cell (strain MG1655) after flow of iso-osmotic media (drug-containing LB with 0 mM sorbitol). Scale bar, 1 μm.

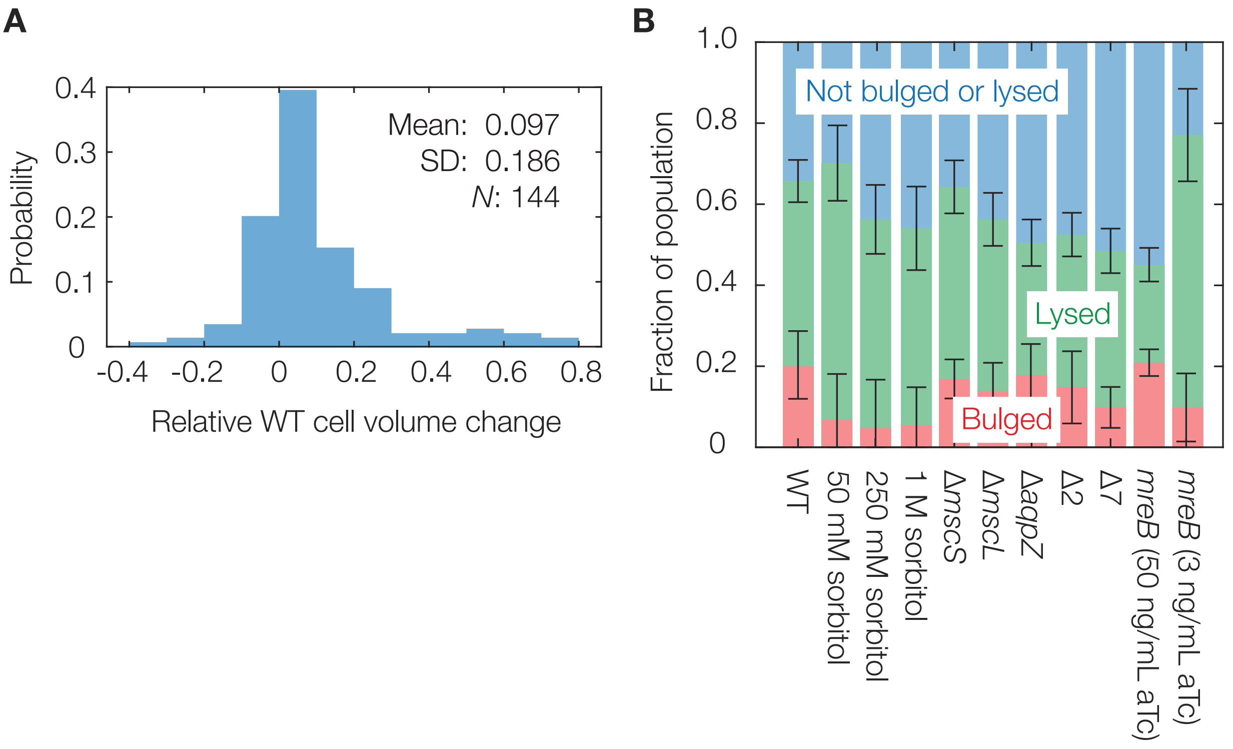

Figure S6. Cell size changes during bulging and bulging fractions in different strains and conditions.
(A) Distribution of relative cellular volume changes from before bulging to immediately before lysis in wild-type *E. coli* (strain MG1655) cells, showing that typical cellular volume changes are limited during bulging. N indicates number of bulged cells. Cellular dimensions were determined as discussed in *Methods*.
(B) Fractions of bulged or lysed cells in different strains and conditions 2 hrs after cephalexin treatment, within each field of view comprising at least 50 cells. Data are representative of two to three biological replicates for each bar, and error bars indicate one standard deviation. *mreB*, *mreB*-titratable strain.

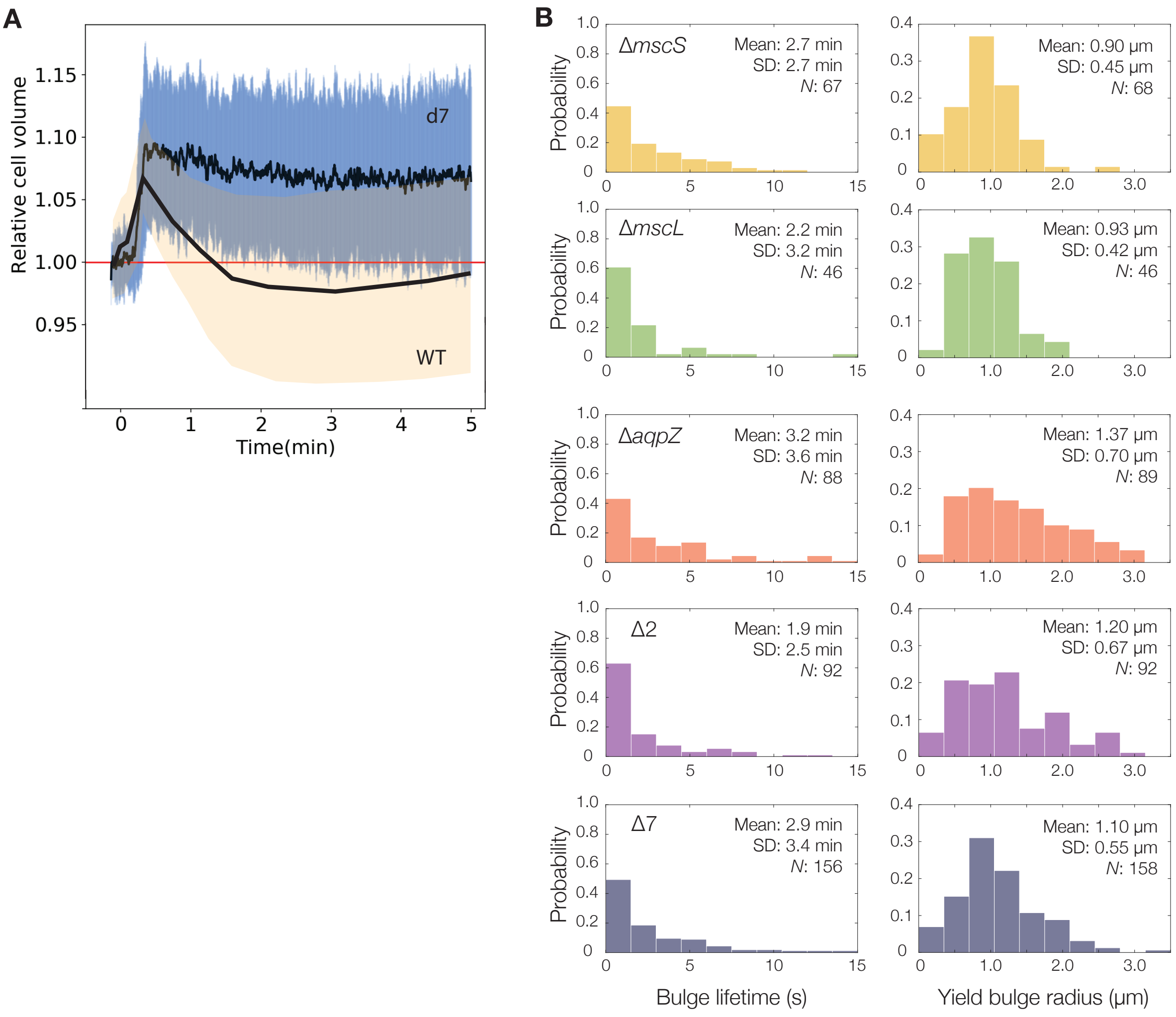

Figure S7. Characterization of MSC mutants.
(A) Average volume response for the relative cell volume of the Δ7 strain upon a 300 mM (460 mOsmol) hypoosmotic shock with NaCl in modified M9 media. The first 5 minutes following the hypoosmotic shock shows no volume recovery that is characteristic of MSC activation, as indicated by the sharp decrease in cellular volume after ∼1 min in Figure 3A of the main text. The black curve shows the average response of individual single cell volumes ($N = 30$), and the shaded region indicates one standard deviation. Images were captured every 0.33 s. Individual cell volume traces were first filtered with a median filter of window size 5 and aligned at the first point of maximum volume expansion. For comparison, measurements of the volume responses of wild-type cells (parent strain BW25113) in the same conditions, adapted from ref. (Buda et al., 2016), are shown.
(B) Distributions of bulge lifetimes and yield bulge radii for MSC mutants. For full timelapses of lysing populations, see Movies S4 and S5.

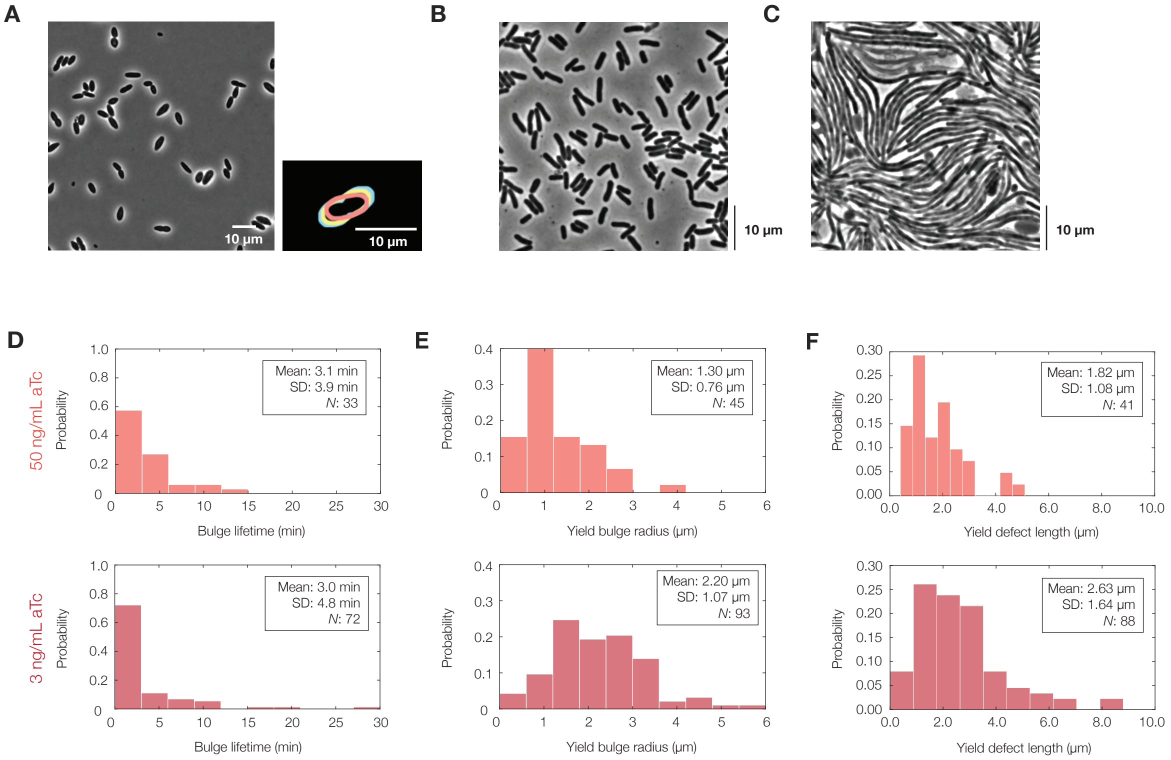

Figure S8. Additional measurements for *mreB*-titratable *E. coli*.
(A) A population of control, untreated *mreB*-titratable cells 2 h after plating on an LB-agarose pad containing 3 ng/mL aTc. The *mreB*-titratable strain is described in previous work (Zheng et al., 2016). (Inset) A contour image corresponding to Figure 4D of the main text.
(B) Phase-contrast image of a population of *mreB*-titratable cells, at an aTc concentration of 50 ng/mL, immediately after cephalexin (50 μg/mL) treatment.
(C) Same as (B), but 2 hrs after cephalexin (50 μg/mL) treatment.
(D-F) Distributions of bulge lifetimes (D), yield bulge radii (E), and yield defect lengths (F) for *mreB*-titratable cells at aTc concentrations of 3 and 50 ng/mL. The yield defect lengths r_d were estimated, according to the model, by the formula $R = r_d \sin\theta$, where R is the yield bulge radius and θ is the subtended angle of the bulge.

Strain	Genotype	Source	Cephalexin MIC
MG1655	F-, λ-, rph-1	Laboratory stock	20 μg/mL
JOE309	MC4100 araD+	Timelapse data from ref. (Yao et al., 2012)	20 μg/mL
BW25113	F-, Δ(araD-araB)567, ΔlacZ4787(::rrnB-3), λ-, rph-1, Δ(rhaD-rhaB)568, hsdR514	Gift from Thomas Bernhardt	20 μg/mL
W3110	F-, λ-, IN(rrnD-rrnE)1, rph-1	Laboratory stock	20 μg/mL
JW2891-2 (Keio Δ*mscS*)	BW25113 ΔmscS775::kan	Gift from Douglas Weibel	20 μg/mL
JW3252-1 (Keio Δ*mscL*)	BW25113 ΔmscL727::kan	Gift from Douglas Weibel	20 μg/mL
JW0859-5 (Keio Δ*aqpZ*)	BW25113 ΔaqpZ776::kan	Gift from Douglas Weibel	20 μg/mL
"Δ2"	BW25113 ΔmscL727::kan, ΔmscS775::kan pWR21	Laboratory of Teuta Pilizota (Buda et al., 2016)	20 μg/mL
"Δ7"	20 μg/mL	Laboratory of Teuta Pilizota (Hegde, 2020)	20 μg/mL
AMB1655	F-, λ-, Δfnr-267, rph-1; G>T gudD2917303, flhDC::IS	Antoine Danchin	Not determined
ZH1 ("*mreB*-titratable")	AMB1655 mreB<>aph, bla:Ptet-tetR-mreB at attB site	Laboratory of Chenli Liu (Zheng et al., 2016)	20 μg/mL (3-50 ng/mL aTc); 20-40 μg/mL (0 ng/mL aTc)

Table S1. Strains used in this study and their cephalexin MICs.

Variable	Value	Reference
**Mechanical model of bacterial cell lysis**		
Cell wall 2D elastic modulus (axial), $Y_x^w (= Y^w)$	0.1 N/m	(Deng et al., 2011; Amir et al., 2014; Tuson et al., 2012)
Cell wall 2D elastic modulus (circumferential), $Y_y^w (= Y^w)$	0.2 N/m	(Deng et al., 2011; Lan et al., 2007)
Inner membrane area-stretch modulus, $K_a^i (= K)$	0.1 N/m	(Sun et al., 2014)
Outer membrane area-stretch modulus, $K_a^o (= K)$	0.1 N/m	(Sun et al., 2014)
Cell wall Poisson's ratio, $\nu_{xy}^w (= \nu^w)$	0.2	(Yao et al., 1999)
Cell wall Poisson's ratio, $\nu_{yx}^w (= \nu^w)$	0.4	$\nu_{yx}^w = Y_y^w \nu_{xy}^w / Y_x^w$
Turgor pressure, p	0.5 atm	(Deng et al., 2011; Cayley et al., 2000)
Number of solute molecules inside a cell, n_s	9.5×10^7	$n_s \approx p[\pi (r_0^w)^2 L_0^w]/kT$
Reference cell wall radius, r_0^w	0.5 μm	This work
Reference cell wall length, L_0^w	10 μm	This work
Reference membrane surface area ratio, γ	1.1	Intermediate value of the range inferred in (Wong and Amir, 2019)
Cell wall defect radius, r_d	0.5 μm	This work
Temperature, T	300 K	This work
**Transport model of solute outflow**		
Viscosity of water, μ	8.9×10^{-4} Pa · s	—
Density of water, ρ	997 kg/m^3	—
Volume occupied per water molecule, m_w	3×10^{-29} m^3	—
Characteristic MSC hole radius, r_c	1.5 nm	(Naismith and Booth, 2012; Buda et al., 2016)
Characteristic transport length across bilayer, L_c	10 nm	This work; membrane thickness estimate from (Phillips et al., 2012)
Characteristic number of gated channels, $\mathcal{N}$	100	(Bialecka-Fornal et al., 2012; Chure et al., 2018)
Membrane hydraulic conductivity, L_p	10^{-12} m^3/N · s	(Sperelakis, 1995; Çetiner et al., 2017)

Table S2. Model parameters for *E. coli* used in this study. Parameter value simplifications are indicated in parentheses.

				Exponential		Bootstrapped CI's	
	Condition	N	Mean	χ^2 test	p-value	LCI	UCI
Bulge lifetime (s)	WT	406	199.35	1	5.2E-11	173.07	233.45
	WT+0 mM sorbitol	48	373.12	1	7.6E-03	280.67	482.94
	WT+50 mM sorbitol	76	1105.1	0	4.6E-01	909.42	1365.1
	WT+250 mM sorbitol	27	1615.2	0	N/A	1159.1	2165.7
	WT+1 M sorbitol	25	2559.5	0	N/A	1791.9	3955.1
	Δ*mscS*	67	161.31	0	4.0E-01	126.25	204.92
	Δ*mscL*	46	130.76	0	1.2E-01	87.69	207.2
	Δ*aqpZ*	88	193.14	0	2.6E-01	153.23	244.81
	Δ2	92	115.39	0	1.6E-02	88.16	151.77
	Δ7	156	171.25	0	9.3E-02	142.81	206.32
	mreB-titratable (50 ng/mL aTc)	33	186.11	0	1.5E-02	120.78	279.98
	mreB-titratable (3 ng/mL aTc)	72	178.45	0	2.5E-02	125.59	267.92
				Lognormal			
Yield bulge radius (μm)	WT	390	0.9	1	1.8E-04	0.86	0.95
	WT+0 mM sorbitol	48	0.91	0	2.3E-01	0.83	1.01
	WT+50 mM sorbitol	67	0.77	1	9.6E-03	0.69	0.88
	WT+250 mM sorbitol	21	1.01	0	N/A	0.89	1.12
	WT+1 M sorbitol	25	1.10	0	N/A	1.00	1.21
	Δ*mscS*	68	0.9	0	1.0E-01	0.8	1.01
	Δ*mscL*	46	0.93	0	4.1E-02	0.82	1.06
	Δ*aqpZ*	89	1.37	0	4.8E-01	1.22	1.52
	Δ2	92	1.2	0	2.5E-02	1.07	1.34
	Δ7	158	1.1	1	1.1E-03	1.02	1.19
	mreB-titratable (50 ng/mL aTc)	45	1.3	0	1.2E-01	1.11	1.55
	mreB-titratable (3 ng/mL aTc)	93	2.2	0	5.6E-02	2	2.44

Table S3. Statistical testing of inferred distributions and calculated confidence intervals. Here N indicates sample size (numbers of bulged cells), LCI and UCI denotes the lower and upper bounds for the 95% confidence interval (CI) for the mean, respectively, and the test used is the χ^2 goodness-of-fit, where 1 indicates rejection of the null hypothesis that the data come from the distributions shown and 0 otherwise. N/A indicates that there are insufficient degrees-of-freedom for this test. The bootstrapped confidence intervals are calculated as detailed in *Methods*.
